# Photoactivatable Nanobody Conjugate Dimerizer Temporally Resolves Tiam1‐Rac1 Signaling Axis

**DOI:** 10.1002/advs.202307549

**Published:** 2024-01-15

**Authors:** Chengjian Zhou, Huiping He, Xi Chen

**Affiliations:** ^1^ Laboratory of Chemical Biology and Frontier Biotechnologies The HIT Center for Life Sciences (HCLS) Harbin Institute of Technology Harbin 150001 P. R. China; ^2^ School of Life Science and Technology Harbin Institute of Technology Harbin 150001 P. R. China

**Keywords:** actin cytoskeleton, apoptosis, chemically induced proximity, chemo‐optogenetic dimerization, lamellipodia, light control, optochemical biology

## Abstract

The precise spatiotemporal dynamics of protein activities play a crucial role in cell signaling pathways. To control cellular functions in a spatiotemporal manner, a powerful method called photoactivatable chemically induced dimerization (pCID) is used. In this study, photoactivatable nanobody conjugate inducers of dimerization (PANCIDs) is introduced, which combine pCID with nanobody technology. A PANCID consists of a nanobody module that directly binds to an antigenic target, a photocaged small molecule ligand, and a cyclic decaarginine (cR_10_*) cell‐penetrating peptide (CPP) for efficient nonendocytic intracellular delivery. Therefore, PANCID photodimerizers also benefit from nanobodies, such as their high affinities (in the nm or pm range), specificities, and ability to modulate endogenous proteins. Additionally it is demonstrated that the nanobody moiety can be easily replaced with alternative ones, expanding the potential applications. By using PANCIDs, the dynamics of the Tiam1‐Rac1 signaling cascade is investigated and made an interesting finding. It is found that Rac1 and Tiam1 exhibit distinct behaviors in this axis, acting as time‐resolved “molecular oscillators” that transit between different functions in the signaling cascade when activated either slowly or rapidly.

## Graphical abstract

Nanobody‐based photoactivatable chemically induced dimerization, known as PANCID, is introduced. PANCID combines the benefits of nanobodies and photoinducible chemical dimerizers, and it is utilized to achieve spatiotemporal control of cellular processes. Specifically, the Tiam1‐Rac1 signaling axis is studied and a scenario in which Tiam1 induces apoptosis is revealed.

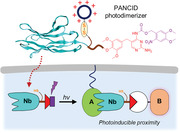

## 1 Introduction

Chemically induced dimerization (CID) or chemically induced proximity (CIP) is a powerful approach to control and study cellular processes based on a proximity‐inducing mechanism.^[^ ^1^ ^]^ To achieve higher spatiotemporal precision, photoactivatable or photo‐responsive CIDs (pCIDs) that contain photocaged ligands have been developed.^[^ ^2^ ^]^ Light‐control offers several advantages, including high temporal precision, high spatial precision, and noninvasiveness, making pCIDs particularly attractive for regulating cellular functions.^[^ ^3^ ^]^ Therefore, these chemo‐optogenetic dimerizers are valuable candidates for studying biological questions.^[^ ^4^ ^]^ Meanwhile, they also serve as indispensable alternatives to classic optogenetic dimerizers,^[^ ^5^ ^]^ complementing each other in terms of origin, photoactivation method, instrumentation requirement, and other factors.

pCIDs were initially created by adding a photocage to an existing noncovalent dimerizer like rapamycin, resulting in photoactivatable CIP molecules.^[^ ^2a^ ^]^ Subsequently, cell‐permeable mono‐covalent^[^ ^4^, ^6^ ^]^ and bi‐covalent^[^ ^7^ ^]^ versions of pCID molecules were introduced, taking advantage of covalent binding to achieve unprecedented spatial precision^[^ ^6^, ^8^ ^]^ due to the resistance to diffusion. More recently, photoswitchable versions that can be activated and deactivated using different wavelengths of light^[^ ^8a^ ^]^ have been introduced to control cellular functions in a switch‐like fashion.^[^ ^8^, ^9^ ^]^ Nevertheless, these covalently binding pCIDs also have certain limitations, including compromised labeling efficiency *in cellulo*, prolonged incubation time, and the need to extensively wash out excessive dimerizers, which can negatively affect induced dimerization due to the so‐called hook effect^[^ ^10^ ^]^ and compromise the optimal photoactivatable dimerization efficacy in biological studies.^[^ ^11^ ^]^ Therefore, it is necessary to design next‐generation photoactivatable dimerizers that are easier to use, eliminate the need for labeling and extensive washing, and yet retain a high affinity dimerization efficacy.

The Tiam1‐Rac1 signaling axis^[^ ^12^ ^]^ is an important signaling cascade that primarily mediates lamellipodia formation. Tiam1 (T‐lymphoma invasion and metastasis‐inducing factor‐1) is a direct upstream factor of Rac1, which activates Rac1 and subsequently leads to lamellipodia formation in live cells. ^[^ ^13^ ^]^ However, evidences suggest that the functions of Tiam1 are more complicated than usually expected. Tiam1 may also activate other factors besides Rac1, resulting in significantly different cellular events.^[^ ^13^ ^]^ Additionally, Rac1 can induce various downstream cellular activities, such as cell protrusion, membrane ruffling, or adhesion spot formation.^[^ ^14^ ^]^ Therefore, the exact regulation of the Tiam1–Rac1 signaling axis in relation to these diverse cellular activities still remains unclear.

In particular, the Tiam1–Rac1 axis has been found to be associated with cell survival, apoptosis, and other functions.^[^ ^15^ ^]^ Studies have shown that Tiam1 depletion can reduce Rac1 activation and trigger apoptosis in specific situations.^[^ ^16^ ^]^ Conversely, evidence suggests that Tiam1 can also increase intracellular reactive oxygen species (ROS) and leads to cell apoptosis.^[^ ^17^ ^]^ Despite Tiam1's involvement in apoptosis, the mechanism underlying its promotion or inhibition of apoptosis remains unclear. To shed light on these questions, we utilized our easy‐to‐use high affinity PANCID system to temporally investigate the Tiam1–Rac1 signaling axis. Our findings reveal that Rac1 and Tiam1 exhibit distinct behaviors in the signaling cascade. Both Tiam1 and Rac1 act as time‐resolved oscillators, leading to different downstream events depending on the speed of activation.

## 2 Results and Discussion

### 2.1 Design, Preparation, and Biochemical Characterization of PANCID

**Figure 1:**
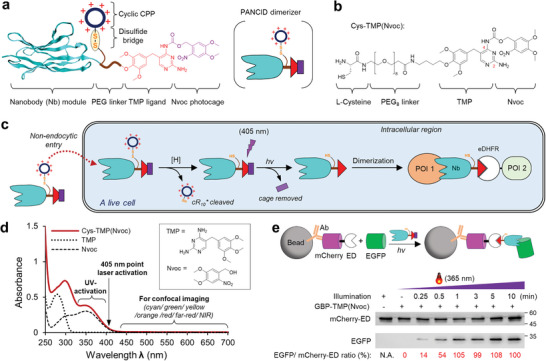
Design, preparation, and biochemical characterizations of cR_10_*‐SS‐GBP‐TMP(Nvoc) (cRGTN). a) Schematic view of the structural elements of a PANCID photodimerizer that consists of a nanobody module, a PEG linker, a Novc‐ caged TMP ligand, and a disulfide bridged cyclic CPP; a symbolic view of a PANCID is given in the right. b) The chemical structure of Cys‐TMP(Nvoc) that features a free cysteine moiety, a PEG_8_ linker, and a Nvoc‐ caged TMP ligand. c) Schematic view of the general working principle of PANCID. d) UV–vis absorption spectra of 100 µM Cys‐TMP(Nvoc) (dark red line) in comparison with TMP and Nvoc (dashed lines) reveal that this photocaged molecule carries a Nvoc photocage (approximately 325–415 nm). e) Pull‐down experiment using mCherry antibody coated magnetic beads that are mixed with a solution of EGFP (1.0 equiv), mCherry‐eDHFR (1.0 equiv.) and GBP‐TMP(Nvoc) (1.5 equiv.) in PBS irradiated with different dosages of 365 nm UV light.

Green fluorescent protein (GFP) and its variants, such as enhanced GFP (EGFP), are widely used fluorescent proteins (FPs) in biology. Therefore, our first objective was to develop a generally applicable PANCID dimerizer called cR_10_*‐SS‐GBP‐TMP(Nvoc), abbreviated as cRGTN, that can induce protein dimerization associated with EGFP fused proteins (**Figure** **1**a). This photoactivable dimerizer consists of a green fluorescent protein binding protein (GBP) nanobody,^[^ ^18^ ^]^ an extended water‐soluble PEG linker, and an *O*‐nitroveratryloxycarbonyl (Nvoc)‐photocaged trimethoprim ligand TMP(Nvoc). The TMP(Nvoc) moiety was introduced using a rationally synthesized Cys‐TMP(Nvoc) building block (Figure 1b). Additionally, a cyclic decaarginine cR_10_* cell‐penetrating peptide (CPP) (Figure S1, Supporting Information) was also attached via a reductively cleavable disulfide bond to enable efficient nonendocytic intracellular delivery. Therefore, cRGTN can easily cross the plasma membrane, release the cR_10_* moiety, induce dimerization between EGFP‐ and eDHFR‐fused proteins, and subsequently control cellular processes through the mechanism of induced proximity (Figure 1c).

cRGTN is assembled through two major steps: The first step involves expressed protein ligation (EPL) to ligate the TMP(Nvoc) photocaged ligand to GBP nanobody; and the second step is disulfidization coupling, which attaches the cyclic cR_10_* module via a disulfide bond (Figure S2a, Supporting Information). To achieve this, the Cys‐TMP(Nvoc) molecule was designed and synthesized in advance (Scheme S1, Supporting Information). Cys‐TMP(Nvoc) features an L‐Cys moiety for EPL, a water‐soluble PEG_8_ linker, and a Nvoc caged TMP ligand at its *N* ^2^‐pyrimidine amine position (Figure 1b). The *N* ^2^‐pyrimidine caging position was validated using first HMBC and subsequent NOESY 2D NMR spectra (Supporting Information). Upon UV or 405 nm light irradiation, the Nvoc photocage can be easily removed to release the free trimethoprim (TMP) ligand. This ligand binds with *Escherichia coli* dihydrofolate reductase (eDHFR) with high affinity (Ki = 1.3 nm), but a much lower affinity (Ki = 4–8 µm) for mammalian DHFR.^[^ ^19^ ^]^ In parallel, a cyclic decaarginine CPP (cR_10_*) was synthesized using standard solid‐phase peptide synthesis from Rink amide resin and obtained with high purity (Figure S1, Supporting Information). Subsequently, Cys‐TMP(Nvoc) was ligated to GBP‐Intein‐His_6_ (**I**) to produce GBP‐TMP(Nvoc) (**III**) dimerizer (Figure S2b,c, Supporting Information). Then, Cys‐cR_10_* was attached to the single free sulfhydryl functional group of GBP–TMP(Nvoc) via disulfidization coupling using Ellman's reagent. A comparison between reducing and nonreducing SDS‐PAGE results revealed that Cys‐cR_10_* has been attached onto GBP‐TMP(Nvoc), and the cR_10_* module can indeed be readily cleaved under reducing conditions (Figure S2d, Supporting Information).

Then, we biochemically characterized cRGTN in terms of light‐induced dimerization. First, we recorded the UV–vis spectra of Cys‐TMP(Nvoc) and compared it with Nvoc cage and TMP ligand. The UV–vis spectra revealed a characteristic absorption peak of the Nvoc cage, ranging from approximately 325 to 415 nm. This suggests the potential for photouncaging using near ultraviolet (UV) light, like the commonly used 365 nm UV light, or a violet point laser, such as a 405 nm laser diode that is widely equipped in most standard confocal microscopes (Figure 1d). Therefore, the photoactivation can be fully compatible with many commonly used FPs,^[^ ^8b^ ^]^ which is advantageous over optogenetic dimerizers that usually cover a broad absorption spectrum in the visible region (e.g., LOV1: 400–500 nm; Cry2: 390–500 nm; PhyB: 550–800 nm). Subsequently, we investigated whether cRGTN enables light‐induced dimerization between EGFP and eDHFR‐fused proteins through a pull‐down assay (Figure 1e, up). In this assay, a mixture of EGFP (1.0 equiv.), mCherry‐eDHFR (1.0 equiv.), and GBP‐TMP(Novc) (1.5 equiv.) in PBS were subjected to gradient dosages of 365 nm UV irradiation. Then the resultant reaction mixtures were incubated with mCherry antibody‐coated magnetic beads, following by thorough‐wash before subjected to Western blot (WB) analysis. According to the pulldown results, the amount of EGFP detected increased with longer illumination time (Figure 1e); on the other hand, in the absence of cRGTN with UV irritation (10 min), no EGFP could be detected. It appears that 1 min of illumination has been sufficient to trigger near maximal degree of dimerization, which is fairly fast. To further validate this light‐triggered dimerization, we employed Förster resonance energy transfer (FRET) method. In the presence of cRGTN, no FRET signal could be detected without photoactivation; whereas a clear FRET signal was observed when photoactivation was applied (Figure S3a,b,Supporting Information). These biochemical studies confirmed that cRGTN induces dimerization between EGFP‐ and eDHFR‐fused proteins upon photoactivation.

### 2.2 cRGTN Induces the Dimerization Between EGFP‐ and eDHFR‐Fused Proteins Inside Living Cells Upon Photoactivation

**Figure 2:**
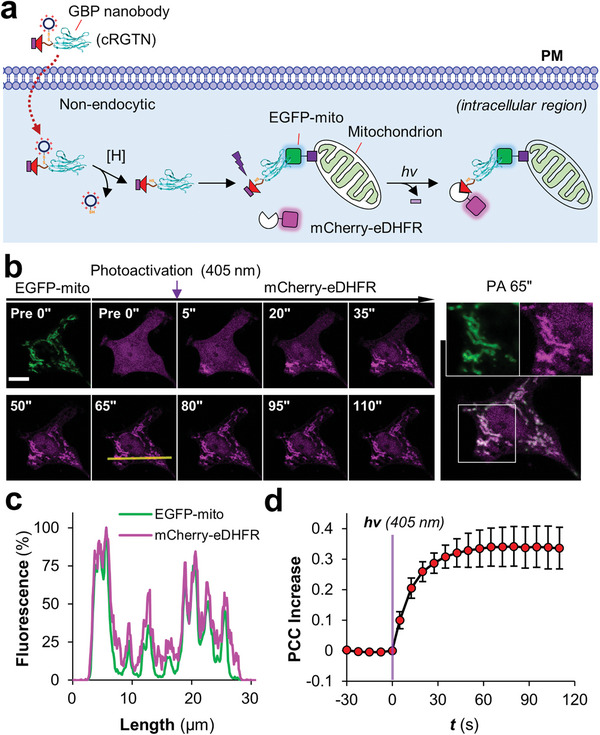
cRGTN induces the dimerization between EGFP and eDHFR upon light illumination. a) Schematic view of the light‐triggered intracellular dimerization assay. b) Live HeLa cells coexpressing mCherry‐eDHFR (magenta, cytosol) and EGFP‐mito (green, mitochondria) treated with cRGTN (24 µm, 1.5 h) were subjected to 405 nm light illumination; time‐lapse confocal micrographs revealed that mCherry‐eDHFR was recruited to mitochondria within seconds and the recruited mCherry‐eDHFR soon distributed throughout all mitochondria in a living cell; scale bar: 10 µm. c) Line profile analysis (left to right) reveals clear colocalization between mCherry‐eDHFR and EGFP‐mito. d) Statistic Pearson's correlation coefficient (PCC) between mCherry and EGFP channels was plotted against time, which revealed rapid time‐dependent recruitment (n = 4 cells).

Afterwards, we tested that whether or not cRGTN can induce protein dimerization inside living cells by light. For this, we first showed that cRGTN readily transduced into live cells in a nonendocytic fashion, even in the presence of the endocytic inhibitor dansylcadaverine (Figure S4, Supporting Information). Using a fluorophore labelled Cys‐cR_10_*, we showed that the cR_10_* moiety was cleaved from cRGTN and localized to nucleolus after entering live cells (Figure S5, Supporting Information). Afterwards, live HeLa cells coexpressing mCherry‐eDHFR (magenta, cytosolic) and EGFP‐mito (green, mitochondria; mito: mitochondrial targeting sequence) were treated with cRGTN (24 µM, 1.5 h) without further washing (**Figure** **2**a). Then cells were directly illuminated with 405 nm light using the default scanner‐operated 405 nm laser module. Shortly after illumination, rapid targeting of mCherry‐eDHFR from cytosol to mitochondria was observed in a time scale of seconds (Figure 2b,c; Movie S1, Supporting Information). The Pearson's correlation coefficient (PCC) between EGFP and mCherry was calculated and then plotted against time revealing a rapid time‐dependent recruitment with *t*_1/2_ of approximately 10 s (Figure 2d). We further showed that cR_10_*‐SS‐GBP without carrying a TMP ligand, and GBP–TMP that does not bear the cR_10_* moiety, are not able to induce protein dimerization inside living cells (Figure S6a–d, Supporting Information) while the uncaged form of cRGTN can directly induce protein translocation (Figure S6e,f, Supporting Information). Finally, we validated the induced dimerization using a transcriptional activation system which confirmed photo‐induced proximity inside living cells (Figure S7, Supporting Information).

Aside from temporal control, we have also demonstrated the ability of cRGTN to enable spatial control of protein dimerization, which is another advantage over chemical inducers of dimerization.^[^ ^3a^ ^]^ According to time‐lapse confocal micrographs, eDHFR‐fused Rac1 was rapidly recruited to a subcellular region of a living HeLa cell where the photoactivation was applied, giving a localized subcellular protein recruitment (Figure S8; Movie S2, Supporting Information). Therefore, these experiments above demonstrated that the cRGTN‐based PANCID system allows no‐wash and photo‐triggered activation of protein–protein dimerization in a spatial and temporal fashion.

### 2.3 Demonstration of the Modularity of PANCID System

We next demonstrated the modular elaboration of cRGTN to expand the application potential in inducing dimerization between different protein/antigen pairs (Figure S9a, Supporting Information). This modular extension concept eliminates the need for synthesizing a new photocaged ligand through time‐consuming multi‐step organic synthesis. Instead, it simply involves the bacterial expression of alternative nanobody‐intein fusions, which is a relatively simple and time‐saving task. To showcase this possibility, we designed and prepared a PANCID dimerizer carrying a mCherry red fluorescent protein binding protein (RBP) nanobody, cR_10_*‐SS‐RBP‐TMP(Nvoc), or cRRTN (**VII**) (Figure S10, Supporting Information). Our results demonstrate that cRRTN efficiently enables light‐triggered protein–protein dimerization by targeting mCherry from the cytosol to the mitochondria (Figure S9b–d; Movie S3, Supporting Information).

Although nanobodies confer noncovalent high binding affinities, there are advantages to using covalent binding, such as localized and permanent labeling, and resistance to non‐covalent diffusions.^[^ ^6^, ^7^, ^8^ ^]^ In this regard, we utilized the SpyCatcher protein tagging technology,^[^ ^20^ ^]^ where the SpyCatcher tag can be seen as a nanobody mimic, with a small size of 15 kDa. SpyCatcher is capable of covalently labeling the SpyTag peptide through the formation of an isopeptide linkage between a Lys of SpyCatcher and the Asp of SpyTag.^[^ ^20^ ^]^ With this in mind, we aimed to establish a covalently localizing PANCID system based on the modular design principle (Figure S11a, Supporting Information). We successfully prepared the cR_10_*‐SS‐SpyCatcher‐TMP(Novc), or cRSTN (**X**) dimerizer using the same preparation pipeline mentioned above (Figure S12, Supporting Information). By using cRSTN (**X**) as the PANCID dimerizer, we were able to achieve light‐controlled protein translocation from the cytosol to the plasma membrane (Figure S11b–d, Supporting Information). These examples highlight the modularity of PANCID system that new photoactivatable dimerizers with unique features can be generated simply by exchanging the nanobody module.

### 2.4 The PANCID Dimerizer cRGTN Temporally Regulates Rac1‐Mediated Lamellipodia Formation

Inspired by the above results, we were motivated to use PANCID photodimerizers to study cell signaling cascades. These photodimerizers are particularly suited for these applications because of their ease of use (no wash) and ability to temporally manipulate protein activities. Cell signaling is a highly dynamic event, and studying it with light‐based techniques allows for better temporal control avoiding prior cellular compensation encountered by other techniques. Our specific interest was in the Tiam1–Rac1 signaling cascade, which is known to trigger cell lamellipodia formation, but many aspects of it are still unclear. Tiam1 is a guanine nucleotide‐exchange factor (GEF) upstream of Rac1, while Rac1 is a key GTPase that regulates actin cytoskeletal dynamics in metazoan cells.^[^ ^12^, ^13^ ^]^ As a small GTPase, Rac1 functions as a molecular switch, cycling between active GTP‐bound and inactive GDP‐bound states. In this study, we utilized a cytosolic and constitutively active form of Rac1, Rac1Q61LΔCAAX (or Rac1*).^[^ ^14^, ^21^ ^]^ Rac1* lacks its C‐terminal CAAX sequence (ΔCAAX), preventing self‐localization to the plasma membrane (PM); therefore, activation of Rac1 can be achieved by bringing Rac1* from cytosol to its functional PM location.

**Figure 3:**
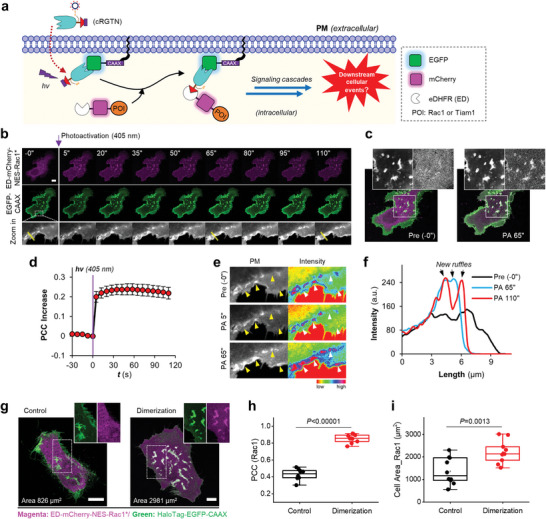
cRGTN temporally dissects the function of Rac1 in inducing lamellipodia formation. a) Schematic view of the experimental design. b) Time‐lapse confocal microscopy images of a live HeLa cell coexpressing eDHFR‐mCherry‐NES‐Rac1Q61LΔCAAX (or ED‐mCherry‐NES‐Rac1*) and EGFP‐CAAX treated with cRGTN (24 µm, 1.5 h) before PA and after PA. c) Enlarged representative snapshots just before PA (Pre ‐0“) and 65 s post PA (PA 65”). d) Statistic PCC co‐localization analysis between EGFP and mCherry channels plotted against time, which reveals extremely fast (< 5“) recruitment of Rac1* to PM (n = 11 cells). e) Intensity coded zoom in images of a subcellular region of a live HeLa cell just before PA (Pre ‐0”), 5 s post PA (PA 5“), and 65 s post PA (PA 65”), which revealed the formation of cell membrane ruffles after PA, and further fluctuation of the ruffle as time pass by. f) Line profile analysis of the ruffle at the subcellular region corresponding to e; the line was drawn as shown in b (yellow line). g) Representative confocal micrographs of live HeLa cells co‐expressing ED‐mCherry‐NES‐Rac1* (magenta) and HaloTag‐EGFP‐CAAX (green) without adding TMP‐Cl dimerizer (control, left) and treated with TMP‐Cl dimerizer (10 µm for 1 h, then wash for 30 min, right). h) Statistical PCC co‐localization analysis (n = 10 cells). i) Statistical analysis of the cell area increase for HeLa cells treated with TMP‐Cl dimerizer (n = 10 cells). Scale bars: 10 µm.

Then, we temporally activated Rac1 using cRGTN by targeting Rac1* to PM with light, which resulted in strong ruffle formation, a characteristic feature of lamellipodia of HeLa cells, in particular upon photoactivation.^[^ ^4^, ^14^ ^]^ The time‐lapse confocal micrographs show that the red‐colored eDHFR‐mCherry‐Rac1* was rapidly recruited to EGFP‐CAAX at the PM within seconds (**Figure** **3**b,c; Movie S4, Supporting Information). We calculated the PCC colocalization between mCherry and EGFP, and plotted the PCC values against time. The curve demonstrates that colocalization increased instantly upon light illumination and reached near‐maximal levels within 5 s, indicating a rapid perturbation of Rac1* (Figure 3d). Subsequently, we observed strong membrane ruffle formation in the EGFP channel (Figure 3e). Line profile analysis of these cell periphery ruffles reveals that a significant membrane ruffle was generated at 65 s post photoactivation (Pre ‐0″ → PA 65″, black to red line), and this membrane ruffle undergone further fluctuation (from PA 65″ → PA 110″, red to cyan line).

We compared our acute light‐perturbation results with a slower perturbation using a chemical inducer of dimerization, called TMP–PEG_5_–Cl, or TMP–Cl (Figure S13a, Supporting Information).^[^ ^10^, ^22^ ^]^ TMP–Cl is a nonphotoactivatable dimerizer, which penetrates live cells, covalently attaches to HaloTag, and then gradually induces protein dimerization after thoroughly wash out of excess of TMP–Cl (Figure S13b,c, Supporting Information). We confirmed that TMP–Cl induces protein dimerization in a slower fashion and eDHFR‐mCherry‐NES‐Rac1* was translocated from cytosol to HaloTag‐EGFP‐CAAX at PM in a time scale of minutes (Figure S13d–f, Supporting Information), orders of magnitude slower than cRGTN‐mediated light perturbation. Interestingly, we did not observe any significant membrane ruffling; instead, we observed a noticeable expansion of the cell surface area. Confocal micrographs further confirmed the targeting of Rac1* to the plasma membrane (Figure 3g,h), accompanied by an expansion of cell areas to over 2000 µm^2^ (Figure 3g,i). Therefore, the acute or gradual activation of Rac1 in live HeLa cells leads to distinct cellular phenotypes.

### 2.5 Th PANCID Dimerizer cRGTN Temporally Regulates Tiam1‐Mediated Signaling Cascade

**Figure 4:**
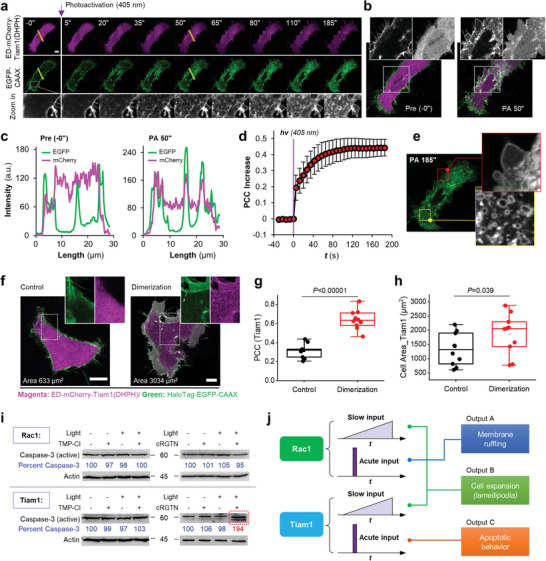
The PANCID dimerization system temporally resolves Tiam1 mediated signalling cascade. a) Time‐lapse micrographs of a representative HeLa cell co‐expressing eDHFR‐mCherry‐Tiam1(DHPH) (magenta, cytosolic) and EGFP‐CAAX (green, PM) treated with cRGTN (24 µm, 1.5 h) before and after photoactivation. b) Enlarged micrographs of the live HeLa cell just before PA (PA ‐0“) and 50 s after PA (PA 50”), show targeting of Tiam1(DHPH) to the plasma membrane after PA, and the appearance of apoptotic phenotypes. c) Line profile analysis (the yellow line in a, from left to right) shows the recruitment of Tiam1(DHPH) to PM after PA. d) Statistical PCC analysis between the two channels plotted against time, shows rapid PM targeting of Tiam1(DHPH) (n = 4 cells). e) Zoom in micrograph of the HeLa cell at 185 s shows a strong apoptotic phenotype with many forming holes on the PM. f) Slow activation of Tiam1(DHPH) using TMP‐Cl which gradually targets eDHFR‐mCherry‐Tiam1(DHPH) from cytosol to PM. g) Statistical PCC analysis between the two channels (n = 10 cells). h) Statistical comparison of the cell area increase after TMP‐Cl induced dimerization (n = 10 cells). i) WB analysis of either Rac1* or Tiam1(DHPH) activated by cRGTN or TMP‐Cl, with or without light illumination confirmed that only acute activation of Tiam1(DHPH) by light can cell apoptosis being induced. j) Schematic summary of the key findings in this study show that slow or acute activation of Rac1 or Tiam1 would lead to rather different cellular events. Scale bars: 10 µm.

Tiam1 is a GEF factor that primarily acts upstream of Rac1 and is involved in the regulation of Rac1‐mediated signaling pathways. However, Tiam1 is also a multifaceted molecule that affects cellular processes through interactions with other signaling proteins,^[^ ^15^ ^]^ rather than solely through its GEF activity. In our study, we investigated the activity of Tiam1 by targeting eDHFR‐mCherry‐Tiam1(DHPH) to the plasma membrane of live HeLa cells using cRGTN with light. Tiam1(DHPH) refers to the Dbl homology (DH) and Pleckstrin homology (PH) domain, which is the GEF domain, of Tiam1. We were able to acutely target Tiam1(DHPH) to the plasma membrane, resulting in the protrusion of the cell periphery and the formation of multiple holes on the plasma membrane (**Figure** **4**a–d **;** Movie S5, Supporting Information). We also included enlarged images of the cell at 185 s postphotoactivation (PA 185″), which clearly revealed the characteristic apoptotic cell phenotypes (Figure 4e). These findings suggest that the acute activation of Tiam1 may be closely associated with the induction of cell apoptosis. And this evidence aligns with previous reports indicating that Tiam1 is involved in apoptotic mechanisms, such as promoting apoptosis in certain cell types^[^ ^23^ ^]^ or regulating Rac1‐independent anoikis.^[^ ^24^ ^]^

For the next experiment, we compared the results above with gradual Tiam1 activation using TMP‐Cl (Figure S14, Supporting Information). Interestingly, we observed that Tiam1 activated by TMP‐Cl did not exhibit any apoptotic phenotypes. Instead, we observed the formation of lamellipodia, which is characterized by cell surface expansion (Figure 4f–h). This finding aligns well with previous studies that use of chemical inducers of dimerization to activate Tiam1.^[^ ^25^ ^]^ Therefore, these comparative results suggest that Tiam1 functions as a temporally resolved oscillator, leading to different downstream events depending on whether it is activated slowly or rapidly.

So far, the involvement of Tiam1 in cell apoptosis has been evidenced, but the exact nature of this phenomenon remains elusive. To further validate above finding, we conducted an additional western blot analysis (Figure 4i) to detect the active form of caspase‐3, which is considered the gold standard for identifying apoptotic cells since all apoptotic pathways converge on caspase‐3 activation in the signaling cascades.^[^ ^26^ ^]^ We performed a total of four group of experiments, activating either Rac1 or Tiam1 with light‐control or with chemical dimerizer control. The results clearly showed that only Tiam1 causes strong increase of active caspase‐3 with light activation. On the other hand, no significant increase in caspase‐3 was observed in all remained groups, including Tiam1 activation by TMP‐Cl or Rac1 activation by either cRGTN with light or TMP‐Cl dimerizer. To further validate the results, we conducted Hoechst staining, which is known to detect apoptotic cells based on the abnormal nucleus shape. The results from Hoechst staining revealed that cell apoptosis was only induced in cells treated with cRGTN in combination with light activation (Figure S15, Supporting Information).

Hence, these biological studies revealed that both Tiam1 and Rac1 function as time‐sensitive “molecular oscillators” that transition between different functions upon acute or slow activation (Figure 4j). We also discovered a condition under which Tiam1 induces cell apoptosis, shedding light on the Janus role of Tiam1 in mediating cellular activities. These findings provide useful information for further in‐depth mechanistic studies on the multifunctional Tiam1 and Rac1 in the Tiam1‐Rac1 signaling.

## 3 Conclusion

In summary, we introduced modular photoactivatable nanobody conjugate inducers of dimerization (PANCID) for chemo‐optogenetic control of signaling cascades. PANCID is a nanobody‐based version of pCID, which offers high affinity and specificity due to the utilization of a nanobody as a key binding module. PANCID dimerizers can be designed in a modular fashion by using the same photoactivatable ligand, Cys‐TMP(Nvoc), along with different readily accessible nanobody fusions. We showcased the preparation of a panel of PANCID photodimerizers carrying a GFP nanobody, mCherry nanobody, or a nanobody mimic (SpyCatcher) enabling covalent localization. PANCID achieves subcellular control of protein positioning and acute recruitment in a time scale of seconds, rendering it a valuable tool to study cellular functions.

Specifically, we employed cRGTN to temporally activate the Tiam1 and Rac1 proteins in the Tiam1‐Rac1 signaling axis. Through comparative studies with the use of slow‐activation chemical dimerizer TMP‐Cl, we demonstrated that both Tiam1 and Rac1 function as time‐resolved “molecular oscillators”, which leads to distinct cellular events when activated acutely or slowly. The gradual activation of Rac1 promotes cell expansion, whereas rapid activation of Rac1 leads to the formation of significant membrane ruffles. On the other hand, slow activation of Tiam1 also activates cell expansion, while acute activation of Tiam1 induces cell apoptosis that is accompanied with the activation of the key apoptotic marker caspase‐3. Hence, we have identified a condition in which Tiam1 can trigger a cell apoptotic response, offering new insights into the multifaced role of Tiam1. Nevertheless, it is important to note that the Tiam1‐Rac1 axis is a complex signaling cascade that involves multiple associated proteins and downstream factors. Therefore, further in‐depth research is necessary to gain a complete understanding of the molecular basis and exact signaling pathway of Tiam1‐mediated apoptosis.

The PANCID methodology may also have a few limitations. Currently, many nanobodies are still unavailable, and generating a new nanobody can be time‐consuming, taking a few weeks. Additionally, the photocaged ligand may require multi‐step organic synthesis for preparation. However, giving that nanobody technology is a rapidly developing field^[^ ^27^ ^]^ and nanobodies are considered the next generation antibody modality, we anticipate that PANCID will be a valuable addition to the toolbox of photoactivatable chemical inducer of dimerization. It will be particularly useful for studying cellular signaling events that require precise temporal control to examine dynamics and avoid potential cellular compensation that could obscure study results.

## Conflict of Interest

This work was also subjected to patent applications.

## Supporting information

Supporting Information

Supplemental Movie 1

Supplemental Movie 2

Supplemental Movie 3

Supplemental Movie 4

Supplemental Movie 5

## Data Availability

The data that support the findings of this study are available in the supplementary material of this article.
