# Supplementary material for: Photoactivatable Nanobody Conjugate Dimerizer Temporally Resolves Tiam1‐Rac1 Signaling Axis
Source: Adv Sci (Weinh). 2024 Jan 15;11(11):2307549. doi: 10.1002/advs.202307549 (PMC10953561; doi:10.1002/advs.202307549)
Supplement: Supplementary file 1 — Supporting Information [file ADVS-11-2307549-s002.pdf]

ADVANCED SCIENCE
Open Access

Supporting Information

for *Adv. Sci.*, DOI 10.1002/advs.202307549

Photoactivatable Nanobody Conjugate Dimerizer Temporally Resolves Tiam1-Rac1 Signaling Axis

*Chengjian Zhou, Huiping He and Xi Chen**

Photoactivatable Nanobody Conjugate Dimerizer Temporally Resolves Tiam1-Rac1 Signaling Axis

Chengjian Zhou, Huiping He and Xi Chen*

Abstract: The precise spatio-temporal dynamics of protein activities play a crucial role in cell signaling pathways. To control cellular functions in a spatiotemporal manner, a powerful method called photoactivatable chemically induced dimerization (pCID) is used. In this study, we introduced photoactivatable nanobody conjugate inducers of dimerization (PANCIDs), which combine pCID with nanobody technology. A PANCID consists of a nanobody module that directly binds to an antigenic target, a photocaged small molecule ligand, and a cyclic decaarginine (cR_{10}*) cell-penetrating peptide (CPP) for efficient non-endocytic intracellular delivery. Therefore, PANCID photodimerizers also benefit from nanobodies, such as their high affinities (in the nM or pM range), specificities, and ability to modulate endogenous proteins. Additionally, we demonstrated that the nanobody moiety can be easily replaced with alternative ones, expanding the potential applications of PANCIDs. By using PANCIDs, we investigated the dynamics of the Tiam1-Rac1 signaling cascade and made an interesting finding. We found that Rac1 and Tiam1 exhibit distinct behaviors in this axis, acting as time-resolved 'molecular oscillators' that transition between different functions in the signaling cascade when activated either slowly or rapidly.

DOI: 10.1002/anie.2021XXXXX

SUPPORTING INFORMATION

Table of Contents

List of Key Abbreviations……………………………………………………………………………………3
Supplementary Figures………………………………………………………………………………………4
Supplementary Movie Captions…………………………………………………………………………..19
Experimental Procedures…………………………………………………………………………………….20
Mammalian cell culture
Plasmid construction
Transfection
Confocal microscopy
Photoactivation
Preparation of the cyclic cell-penetrating peptide Cys-cR_{10}*
Föster resonance energy transfer (FRET)
UV-Vis absorption analysis
Pull-down and WB analysis
Protein expression and purification
Stepwise protocol for assembly of PANCIDs
Image analysis and statistics
Organic Synthesis…………………………………………………………………………………………….23
General
Synthesis of Cys-PEG_8-TMP(Nvoc) (**1**) and related key intermediates
Synthetic scheme toward Cys-TMP(Nvoc)
NMR Spectra…….……………………………………………………………………………………………26
HR-MS Spectra…….…………………………………………………………………………………………38
Author Contributions…………………………………………………………………………………………43

List of Key Abbreviations

cRGTN: cR_{10}*-SS-GBP-TMP(Nvoc) photodimerizer
cRRTN: cR_{10}*-SS-RBP-TMP(Novc) photodimerizer
cRSTN: cR_{10}*-SS-SpyCatcher-TMP(Nvoc) photodimerizer
GBP: green fluorescent protein binding protein nanobody
RBP: mCherry red fluorescent protein binding protein nanobody
Nb: nanobody
ED or **eDHFR**: *E. coli.* dihydrofolate reductase
HT: HaloTag
TMP: trimethoprim
PA: Photoactivation
ROP: region of photoactivation
CIP: chemically induced proximity
CID: chemically induced dimerization
pCID: photoactivatable chemically induced dimerization
PANCID: photoactivatable nanobody conjugate induced dimerization
CPP: cell-penetrating peptide

Supplementary Figures

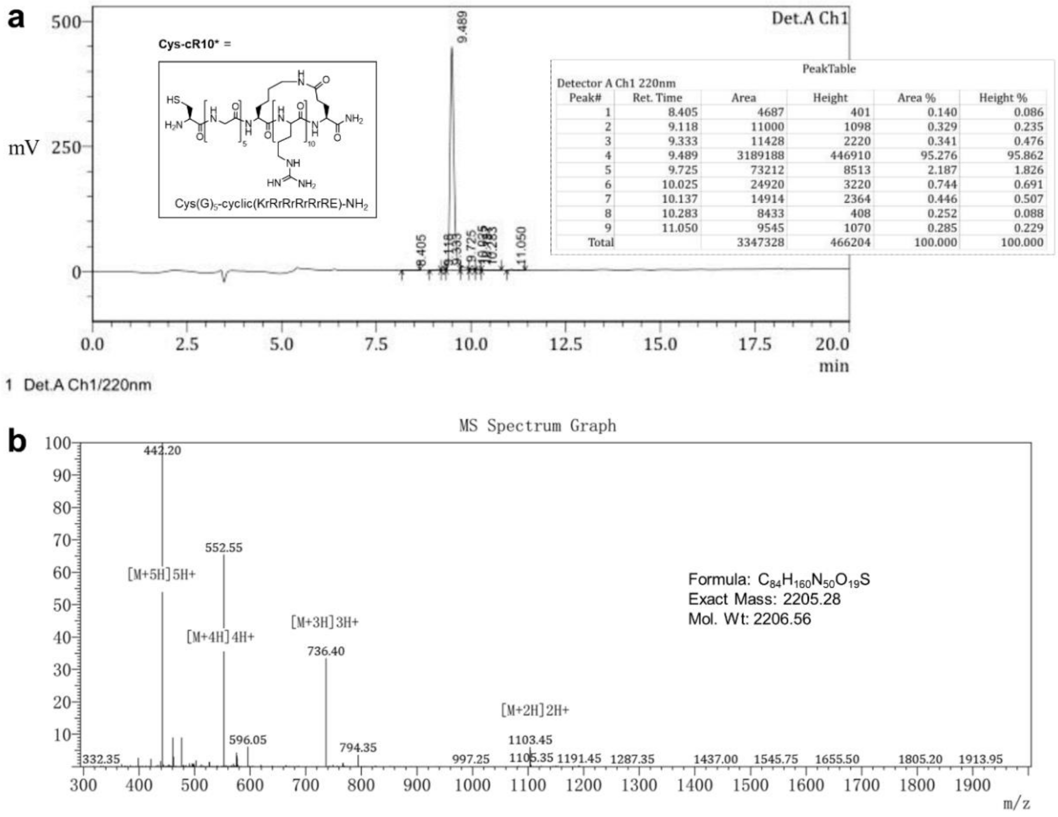

PeakTable

Detector A Ch1 220nm

Peak#	Ret. Time	Area	Height	Area %	Height %
1	8.405	4687	401	0.140	0.086
2	9.118	11000	1098	0.329	0.235
3	9.333	11428	2220	0.341	0.476
4	9.489	3189188	446910	95.276	95.862
5	9.725	73212	8513	2.187	1.826
6	10.025	24920	3220	0.744	0.691
7	10.137	14914	2364	0.446	0.507
8	10.283	8433	408	0.252	0.088
9	11.050	9545	1070	0.285	0.229
Total		3347328	466204	100.000	100.000

Figure S1. Characterization of Cys-cR_{10}* by HPLC and mass spectrometry. **a**, HPLC characterization of Cys-cR_{10}* reveals a high purity of 95.3 %. **b**, ESI-MS characterization of Cys-cR_{10}* reveals correct mass peaks: $C_{84}H_{160}N_{50}O_{19}S$, exact mass: 2205.28, M.W.: 2206.56; found *m/z* 1103.45 $[M+2H]^{2+}$, *m/z* 442.20 $[M+5H]^{5+}$.

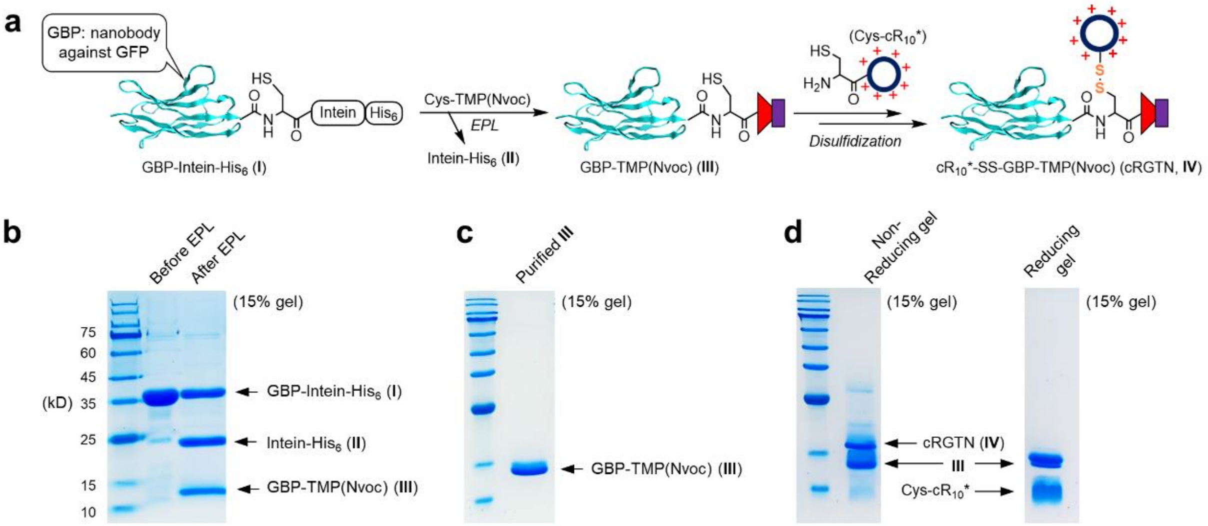

Figure S2. Preparation and SDS-PAGE characterization of cRGTN (**IV**) and its intermediates. **a**, Preparation of a PANCID dimerizer cR_{10}*-SS-GBP-TMP(Nvoc) (cRGTN, **IV**) that invovles two major steps, namely expressed protein ligation (EPL) and disulfidization coupling using the Ellman's reagent (5,5′-dithio-bis-(2-nitrobenzoic acid), DTNB). **b**, SDS-PAGE analysis revealed that GBP-Intein-His_6 (**I**) coupled with Cys-TMP(Nvoc) to create GBP-TMP(Nvoc) (**III**) along with the cleaved Intein-His_6 (**II**) tag. **c**, SDS-PAGE analysis revealed that GBP-TMP(Nvoc) (**III**) can be readily purified via one-step reverse Ni-NTA column chromatography. **d**, Non-reducing (left) and reducing (right) SDS-PAGE analysis of cRGTN (**IV**) revealed that cR_{10}* moiety has been attached to the GBP nanobody, and can be readily detached under reducing conditions. Quantification of the percent of cRGTN via measuring the band intensity by ImageJ gives 53 % of cRGTN in the final product.

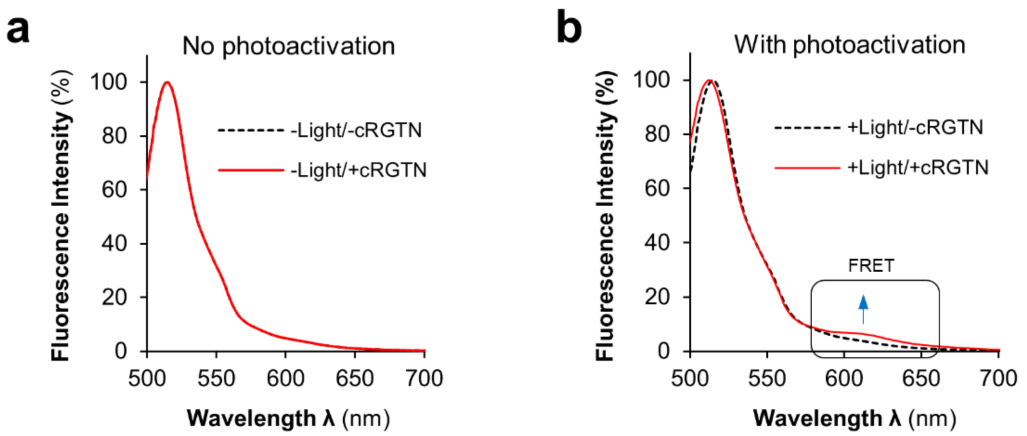

Figure S3. Spectra FRET analysis further validates that cRGTN induces the interaction between EGFP (donor) mCherry-eDHFR (acceptor) upon UV (365 nm) illumination. **a**, Without photoactivation, no FRET signal could be detected with or without cRGTN, suggesting that cRGTN itself does not trigger protein-protein dimerization. **b**, With photoactivation, a clear FRET signal was detected only in the presence of cRGTN, suggesting light-triggered protein-protein dimerization. The maximal fluorescent intensities in the curves were normalized, and more detailed description of FRET experiment was given in the Methods section.

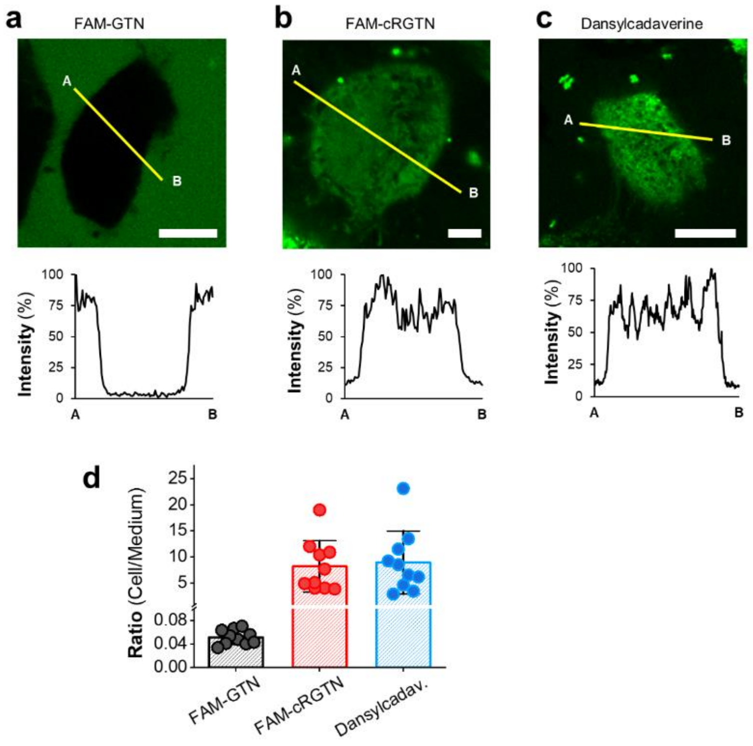

Figure S4. cRGTN enters live cells in a non-endocytic fashion. **a**, FAM labeled GBP-TMP(Nvoc) (i.e. FAM-GTN, 24 μM, 90 min), does not enter living cells, suggesting that GBP-TMP(Nvoc) itself is not cell permeable without carrying cR_{10}*. **b**, FAM labeled cRGTN (i.e. FAM-cRGTN, 24 μM, 90 min) readily enters live cells, suggesting that cRGTN is cell-permeable. **c**, In the presence of the endocytosis inhibitor, dansylcadaverine (50 μM), FAM-labeled cRGTN (24 μM, 90 min) still readily enters live cells without forming endocytic puncta, suggesting a non-endocytic cell-penetrating mechanism. **d**, Statistical quantification of the fluorescence intensity inside the nucleus versus in the culture medium further validated the entry of cRGTN into live HeLa cells. Scale bars: 5 μm.

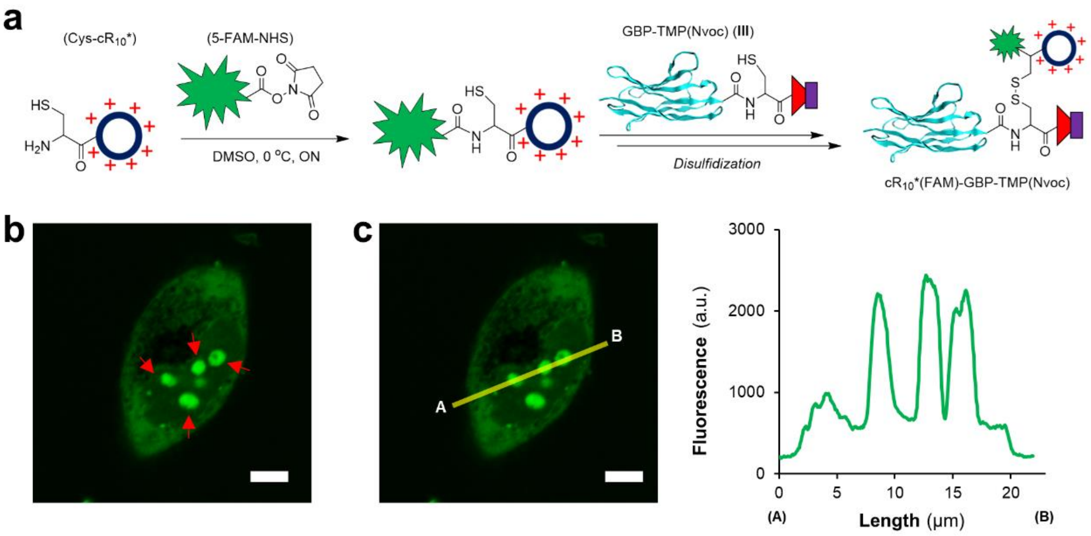

Figure S5. A FAM labeled cR_{10}*-coupled version of cRGTN, i.e. cR_{10}*(FAM)-GBP-TMP(Nvoc) shows that the cR_{10}* was cleaved inside the living cell and localized to the nucleolus. **a**, The preparation scheme shows the reaction conditions and reagents used for the generation of cR_{10}*(FAM)-GBP-TMP(Nvoc). **b**, Representative confocal micrograph shows that cR_{10}*(FAM)-GBP-TMP(Nvoc) (24 μM, 90 min) treated live HeLa cell shows that FAM labeled Cys-cR_{10}* is localized to the nucleolus (see red arrows), suggesting that the cR_{10}* moiety has been cleaved from the PANCID dimerizer. **c**, Line profile analysis further shows the nucleolus localization of cleaved Cys-cR_{10}*. Scale bars: 5 μm.

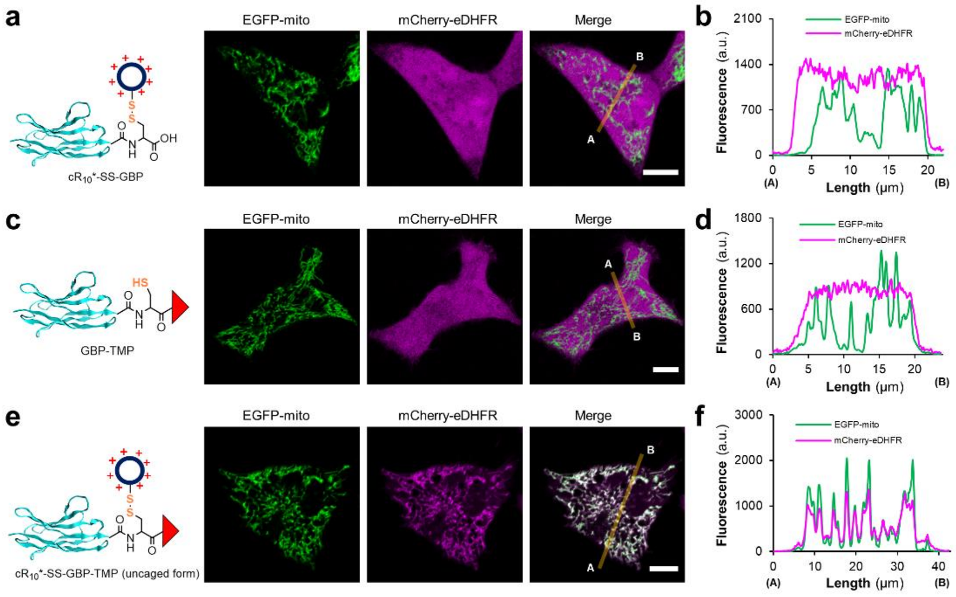

Figure S6. Control experiments show that cR_{10}*-SS-GBP and GBP-TMP(Nvoc) do not induce protein dimerization while cR_{10}*-SS-GBP-TMP, *i.e.* the uncaged form of cRGTN, induces protein dimerization inside living cells. **a**, Representative confocal micrographs show that cR_{10}*-SS-GBP that does not carry a TMP module does not induce protein dimerization inside live HeLa cells; cR_{10}*-SS-GBP was readily prepared via the same pipeline using L-Cys instead of Cys-TMP(Nvoc). **b**, Line profile analysis for the orange line drawn in (**a**). **c**, Representative confocal micrographs show that GBP-TMP that does not carry the cR_{10}* module does not induce protein dimerization inside live HeLa cells suggesting the essential role of cR_{10}* peptide for intracellular delivery; GBP-TMP was readily generated via esterase treatment of GBP-TMP(Nvoc) to remove the Nvoc caging group. **d**, Line profile analysis for the orange line drawn in (**c**). **e**, Representative confocal micrographs show that cR_{10}*-SS-GBP-TMP, i.e. the uncaged form of cRGTN, induces strong protein dimerization inside live HeLa cells; this uncaged form of cRGTN was readily generated via coupling between GBP-TMP with Cys-cR_{10}*. **f**, Line profile analysis for the orange line drawn in (**e**). Scale bars: 10 μm.

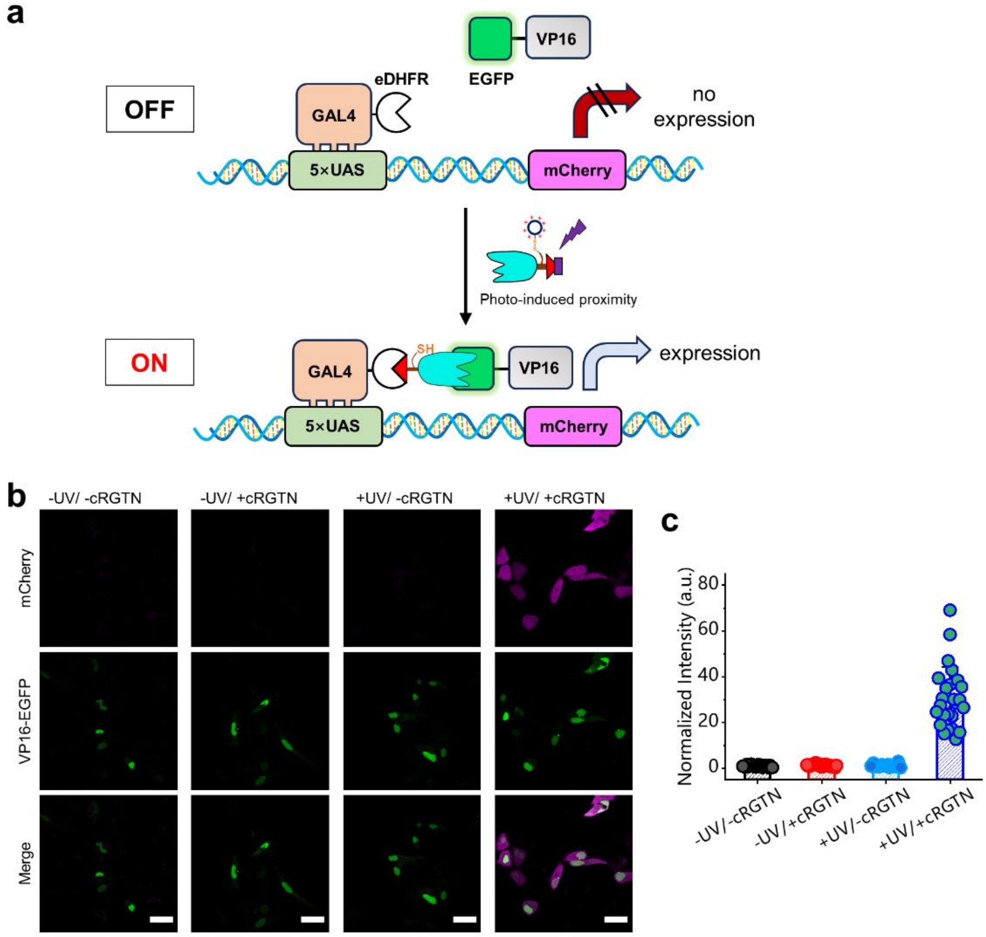

Figure S7. A transcriptional activation system was used to further validate the photo-induced proximity of PANCID. **a**, Schematic view of the transcriptional activation system: Live HeLa cells were transfected with the indicated three plasmids, pUAS-driven mCherry, VIP16-EGFP, and GAL4-eDHFR; PANCID-based photo-induced proximity will target the transcriptional factor GAL4 to UAS and subsequently activate the expression of mCherry fluorescent protein. **b**, Representative confocal micrographs revealed that only in the presence of cRGTN (24 μM) and UV light activation (365 nm, 5 min) will mCherry expression can be induced. **c**, Statistical quantification of the fluorescence intensity of mCherry (n= 25 cells, normalized to the group of -UV/ -cRGTN). Scale bars: 50 μm.

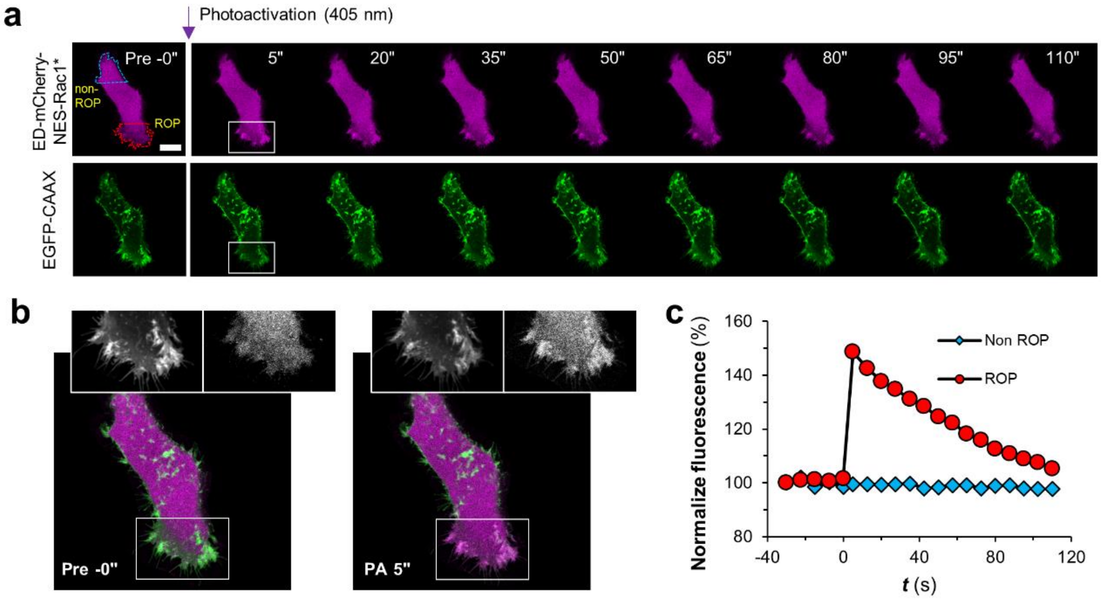

Figure S8. cRGTN also enables spatial control of protein dimerization within a subcellular region of a live cell. **a**, Live HeLa cells coexpressing eDHFR-mCherry-NES-Rac1* (magenta, cytosolic; NES: nucleus exporting signal) and EGFP-CAAX (green, PM) were treated with cRGTN (24 μM, 90 min) and subsequently, photoactivation (PA) was applied within the specified subcellular region of this cell (within red dashed area); time-lapse confocal micrographs were recorded which revealed local recruitment of eDHFR-mCherry-NES-Rac1* to this area. Scale bar: 10 μm. **b**, Zoom in images of this cell (white box in **a**) before photoactivation (Pre -0") and 5 s post photoactivation (PA 5"), which revealed more detailed recruiting processes. **c**, The normalized fluorescence intensity of mCherry at the **r**egion **o**f **p**hotoactivation (ROP, red dashed line circled area in **a**) vs a non-ROP (cyan dashed line circled area in **a**) were plotted against time, which revealed that eDHFR-mCherry-NES-Rac1* was only spatially recruited to this subcellular region but not non-photoactivation region (non-ROP).

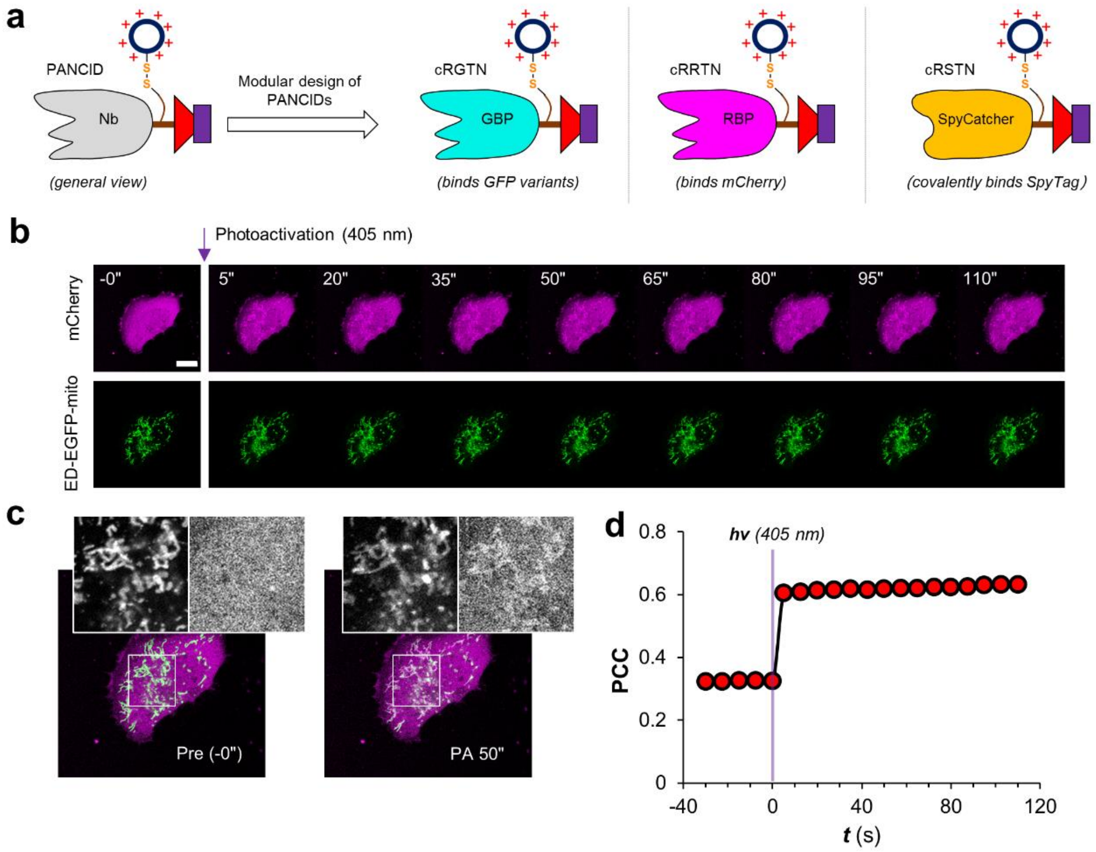

Figure S9. Modular design of different PANCID dimerizers for light-control of protein-protein dimerization inside living cells. **a**, Schematic view of the modular design principle, in which the nanobody could be readily exchanged to alternative nanobody/nanobody mimic using the sample preparation pipeline. **b**, HeLa cells coexpressing mCherry (magenta, cytosol) and eDHFR-EGFP-mito (green, mitochondria) were treated with 24 μM concentration of cR_{10}*-SS-RBP-TMP(Nvoc), or cRRTN, for 90 min in advance. Afterwards, photoactivation (405 nm laser diode) was applied to a particular cell, and then time-lapse confocal microscopic images were recorded, which revealed acute recruitment of mCherry from cytosol to mitochondria in a time scale of seconds. Scale bar: 10 μm. **c**, Representative zoom in images of a cell before (Pre -0") and after (PA 50") photoactivation. **d**, Pearson's correlation coefficient (PCC) analysis between the two channels were calculated and further plotted against time, revealing an acute time-dependent dimerization. **Abbreviations:** cRGTN: cR_{10}*-SS-GBP-TMP(Novc); cRSTN: cR_{10}*-SS-SpyCatcher-TMP(Nvoc); ED: eDHFR; PA, photoactivation; RBP: mCherry red fluorescent protein binding protein; mito: mitochondria targeting peptide sequence.

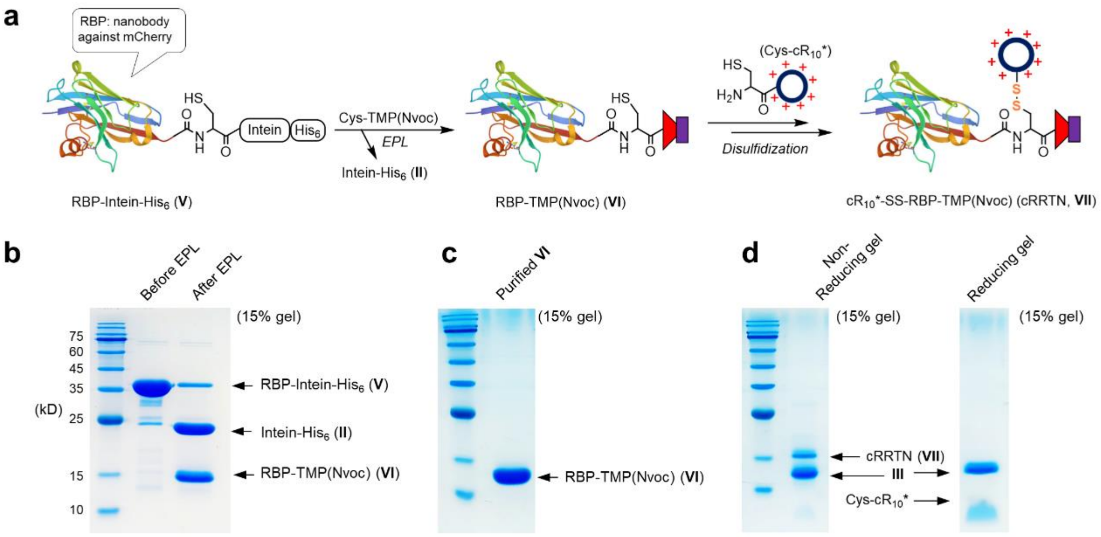

Figure S10. Preparation and SDS-PAGE characterization of cRRTN (**VII**) and its intermediates. **a**, Schematic view of the preparation scheme. **b**, SDS-PAGE analysis revealed that RBP-Intein-His$_6$ (**V**) coupled with Cys-TMP(Nvoc) to create RBP-TMP(Nvoc) (**VI**) along with the cleaved Intein-His$_6$ (**II**) tag. **c**, SDS-PAGE analysis revealed that RBP-TMP(Nvoc) (**VI**) can be readily purified via one-step reverse Ni-NTA column chromatography. **d**, Non-reducing (left) and reducing (right) SDS-PAGE analysis of cRRTN (**VII**) revealed that cR$_{10}$* moiety has been attached to the RBP nanobody, and can also be readily detached under reducing conditions.

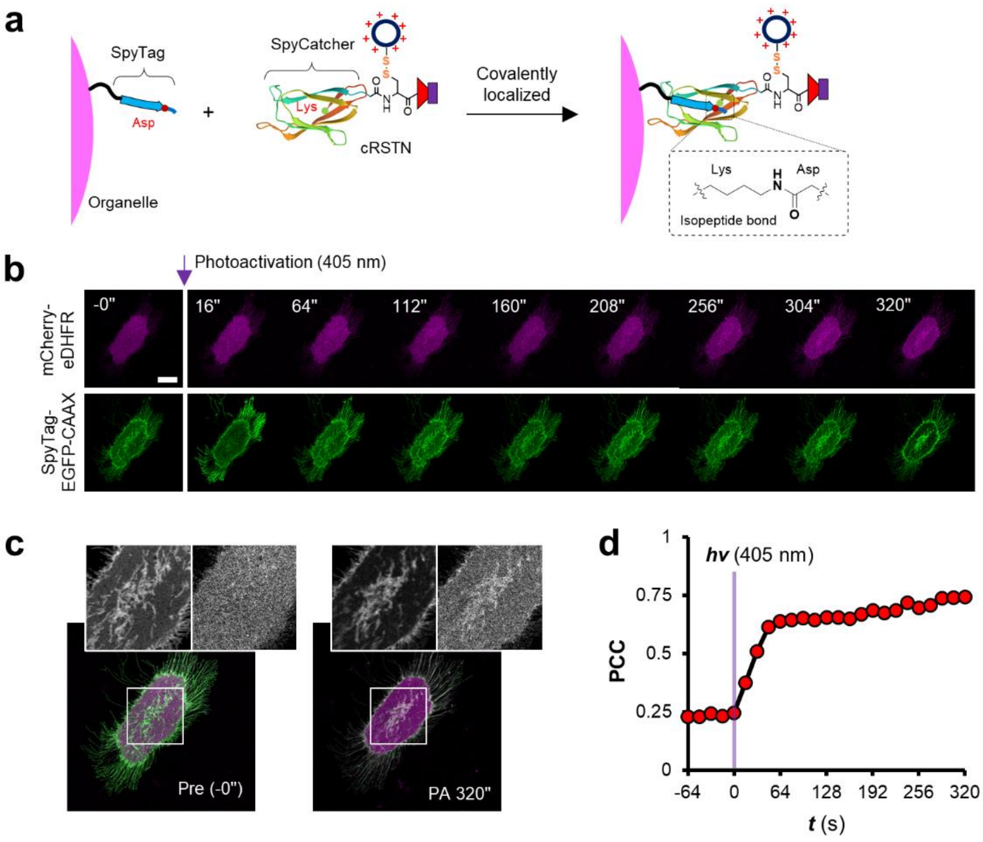

Figure S11. SpyCatcher-containing PANCID photodimerizer enables covalently localized light-control of protein-protein dimerization inside living cells. **a**, Schematic view of the covalently localizing cR_{10}*-SS-SpyCatcher-TMP(Nvoc), or cRSTN, photodimerizer. The modularly designed cRSTN is a covalent version of PANCID dimerizer that could react with SpyTag through the formation of a covalent isopeptide linkage between a Lys of SpyCatcher and the Asp of SpyTag. **b**, Time-lapse confocal microscopic images revealed that cRSTN enabled light-induced dimerization between SpyTag and eDHFR inside living cells; scale bar: 10 μm. **c**, Zoom in images of the live HeLa cell before photoactivation (Pre -0") and after photoactivation (PA 320"). **d**, Pearson's correlation coefficient (PCC) between the two channels were plotted against time showing a time-dependent plasma membrane (PM) targeting of mCherry-eDHFR after light activation.

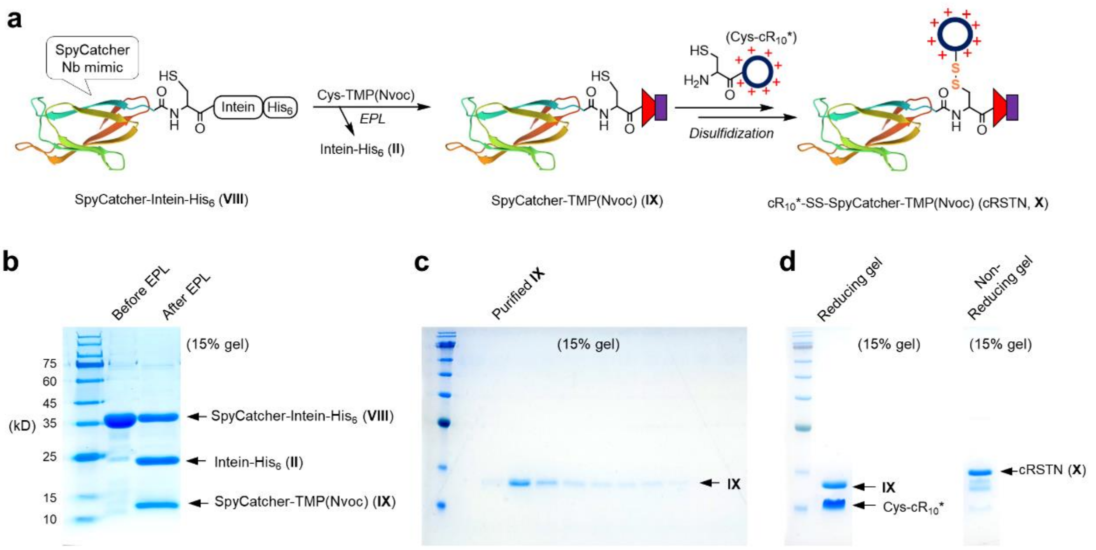

Figure S12. Preparation and SDS-PAGE characterization of cRSTN (**X**) and its intermediates. **a**, Schematic view of the preparation scheme. **b**, SDS-PAGE analysis revealed that SpyCatcher-Intein-His$_6$ (**VIII**) coupled with Cys-TMP(Nvoc) to create SpyCatcher-TMP(Nvoc) (**IX**) along with the cleaved Intein-His$_6$ (**II**) tag. **c**, SDS-PAGE analysis revealed that SpyCatcher-TMP(Nvoc) (**IX**) can be readily purified via one-step reverse Ni-NTA column chromatography without any further purifications. **d**, Reducing (left) and non-reducing (right) SDS-PAGE analysis of cRSTN (**X**) revealed that cR$_{10}$* moiety has been attached to SpyCatcher, and can also be readily detached under reducing conditions.

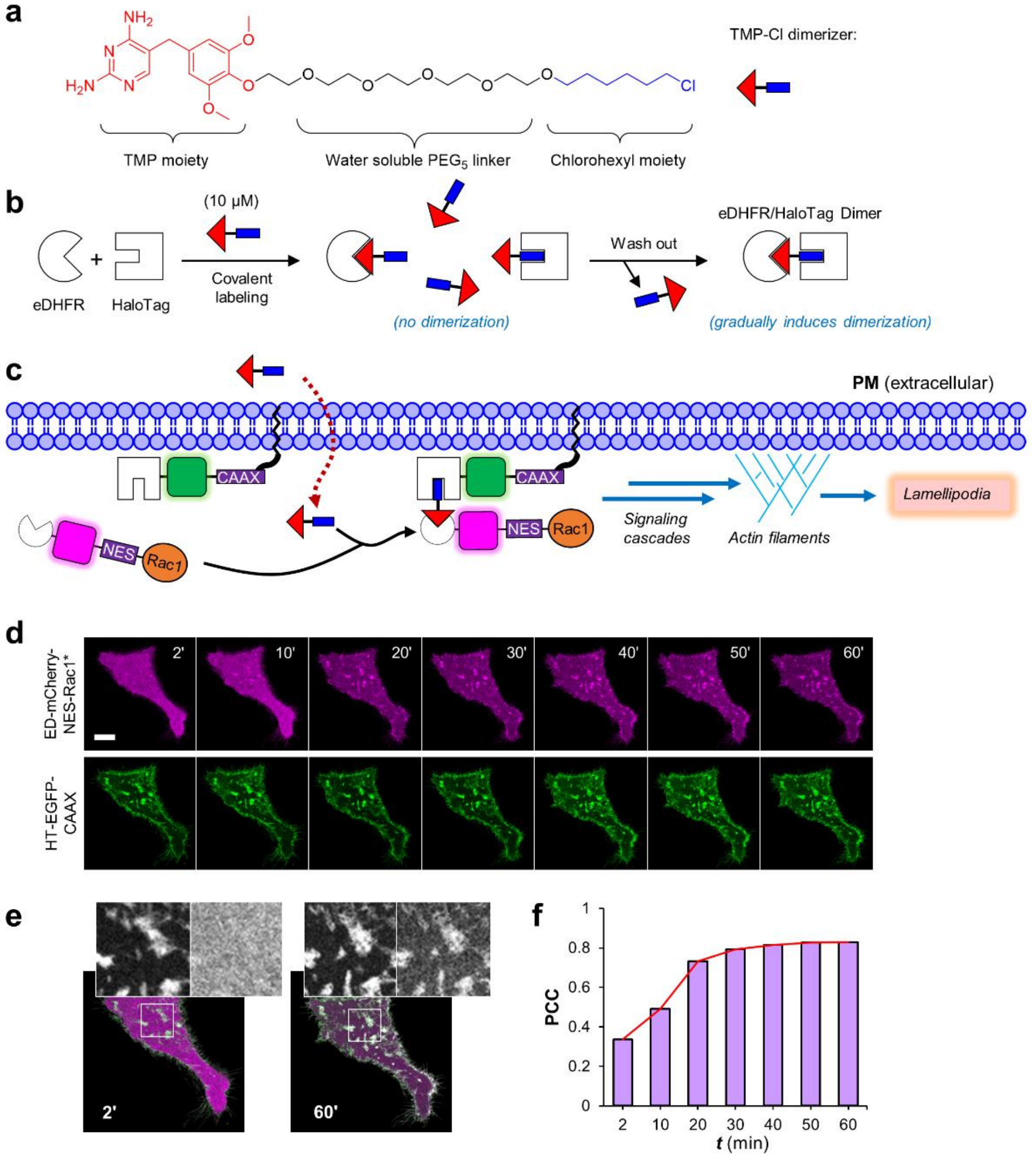

Figure S13. TMP-Cl slowly induces the recruitment of eDHFR-mCherry-NES-Rac1* from cytosol to PM after wash out of excessive TMP-Cl. **a**, Chemical structural the chemical inducer of dimerization TMP-Cl which features a trimethoprim (TMP) ligand for binding with eDHFR, a water soluble PEG_5 linker and a chlorohexyl ligand for covalently binding with HaloTag. **b**, Schematic view of TMP-Cl induced dimerization between eDHFR and HaloTag (HT), in which elevated concentration of TMP-Cl (10 µM, 1 h) is first added to cell culture for covalently anchoring this dimerizer to HaloTag but without inducing any dimerization as TMP-Cl occupies the binding pocket of eDHFR and inhibits the dimerization; after thorough wash out of TMP-Cl, dimerization will be gradually induced. **c**, Schematic view of the mechanism of TMP-Cl induced recruitment of eDHFR-mCherry-NES-Rac1* to the plasma membrane (PM). **d**, Time-lapse confocal micrographs reveal that eDHFR-mCherry-NES-Rac1* was gradually recruited to the plasma membrane after thorough wash out of TMP-Cl; scale bar: 10 µm. **e**, Zoom in images of the cell 2 min post wash out (2') and 60 min post wash out (60'), which reveal more details of the recruitment. **f**, Pearson's correlation coefficient (PCC) between the two channels were plotted against time, revealing a time-dependent translocation in a time scale of minutes. **Abbreviations:** ED: eDHFR; NES: nucleus exporting signal; HT: HaloTag.

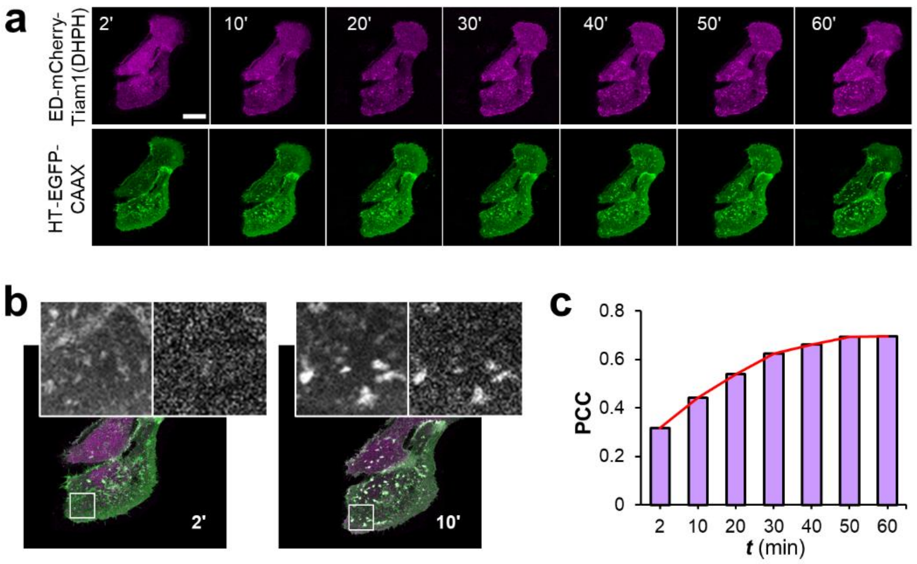

Figure S14. TMP-Cl slowly induces the recruitment of eDHFR-mCherry-Tiam1(DHPH) from cytosol to PM in a time scale of minutes. **a**, Live HeLa cells coexpressing eDHFR-mCherry-Tiam1(DHPH) (magenta, cytosol) and HaloTag-EGFP-CAAX (green, PM) were treated with TMP-Cl dimerizer (10 μM, 1 h) in advance. Afterwards, the cells were thoroughly washed by PBS and then time-lapse confocal micrographs were recorded which revealed that eDHFR-mCherry-Tiam1(DHPH) was gradually recruited from cytosol to PM in a time scale of minutes; scale bar: 10 μm. **b**, Representative zoom-in images of the cell show more details of the recruitment process. **c**, Pearson's correlation coefficient (PCC) analysis between the two channels were calculated and plotted against time, revealing a time-dependent translocation of Tiam1(DHPH) from cytosol to PM. **Abbreviations:** ED: eDHFR; HT: HaloTag; DHPH: Dbl homology (DH) and Pleckstrin homology (PH) domain.

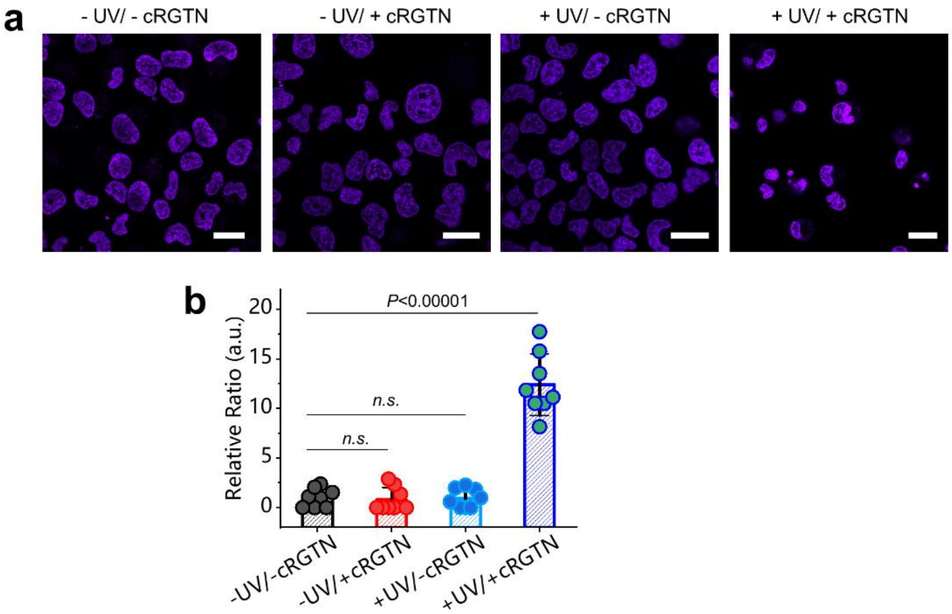

Figure S15. Hoechst 33342-based staining for the evaluation of apoptotic induction. **a**, Representative confocal micrographs of live HeLa cells coexpressing EGFP-CAAX and eDHFR-mCherry-Tiam1(DHPH) labelled with Hoechst 33342 (2.5 μg·ml^{-1}, 20 min) and apoptotic cells will detected which exhibit abnormal nucleus phenotypes. +cRGTN condition: 24 μM, 90 min; +UV condition: 365 nm light (8 W, 5 min). **b**, Statistical quantification (n=8 fields) of the relative apoptotic cell ratios (relative to –UV/–cRGTN group); Student's *t*-test was used; *n.s.*: non-significant. Scale bar: 25 μm.

Supplementary Movie Captions

Movie S1: Photoactivation was globally performed with cRGTN (24 μM, 1.5 h) treated HeLa cell coexpressing mCherry-eDHFR (magenta, cytosolic) and EGFP-mito (green, mitochondria), which revealed acute recruitment of mCherry-eDHFR from cytosol to mitochondria in a time scale of seconds. Related to **Figure 2**.

Movie S2: Photoactivation was locally performed with cRGTN (24 μM, 1.5 h) treated HeLa cell coexpressing eDHFR-mCherry-NES-Rac1* (magenta, cytosolic) and EGFP-CAAX (green, PM), which revealed local recruitment of eDHFR-mCherry-NES-Rac1* from cytosol to PM in less than 5 seconds. Related to **Figure S5**.

Movie S3: Photoactivation was globally performed with cRRTN (24 μM, 1.5 h) treated HeLa cell coexpressing mCherry (magenta, cytosolic) and eDHFR-EGFP-mito (green, mitochondria), which revealed acute recruitment of mCherry from cytosol to mitochondria in less than 5 seconds. Related to **Figure S6**.

Movie S4: Photoactivation was globally performed with cRGTN (24 μM, 1.5 h) treated HeLa cell coexpressing eDHFR-mCherry-NES-Rac1* (magenta, cytosolic) and EGFP-CAAX (green, PM), which revealed acute recruitment of eDHFR-mCherry-NES-Rac1* from cytosol to PM in less than 5 seconds. Related to **Figure 3**.

Movie S5: Photoactivation was globally performed with cRGTN (24 μM, 1.5 h) treated HeLa cell coexpressing eDHFR-mCherry-Tiam1(DHPH) (magenta, cytosolic) and EGFP-CAAX (green, PM), which revealed acute recruitment of eDHFR-mCherry-Tiam1(DHPH) from cytosol to PM in a time scale of seconds. Related to **Figure 4**.

Experimental Procedures

Mammalian cell culture
HeLa (Cat# CL-0101) cells were obtained from Procell Life Science & Technology Co., Ltd. (Wuhan, P.R. China), short tandem repeat (STR) identified and proven to be HIV-1, HBV, HCV, mycoplasma, and other microorganisms free before culturing. Other reagents such as full DMEM (Dulbecco's modified Eagle's medium) and PBS (phosphate buffered saline) were also confirmed to be mycoplasma free before usage. Cell culture were maintained at 37°C under 5 % CO_2 in high glucose (4.5 $g{\cdot}L^{-1}$) DMEM (Cat# SH30243.01, HyClone) containing 4 mM L-glutamine and sodium pyruvate and supplemented with additional 10 % fetal bovine serum (FBS, Cat# SV30087.03, HyClone), 1 % non-essential amino acid (NEAA, 100×), and 1 % penicillin-streptomycin (100×). Trypsin-EDTA (Cat# SH30042.01, HyClone) and PBS (Cat# SH30256.01, HyClone) were used in subculturing. HeLa cells were subcultivated in a ratio of 1:5~10.

Plasmid construction
Plasmid vectors, such as pTXB1, pET28a(+) , EGFP-C1 and EGFP-N1 were obtained from commercial vendors. These parental vectors may be further engineered, such as introducing a His_6- or His_8- affinity tag, insertion of a TEV (ENLYFQ↓G) protease cleavage site, alternation of restriction cleavage sits, or replacing EGFP by other fluorescent proteins, e.g. mCherry, etc. to give modified versions of the parental vector for cloning. Subcloning, Gibson cloning, or modified Gibson cloning methods were employed to construct the desired plasmids. For subcloning, fragments of interest were directly cut from the parent plasmid using appropriate restriction enzymes, or amplified by PCR from plasmids containing the desired genes using hyPerFUsion high-fidelity polymerase (Cat# 1032, APExBIO), gel purified, digested with restriction enzymes and purified again. The gene fragments were ligated into appropriate vectors using T4 DNA ligase. Multiple fragments were assembled by stepwise subcloning or one-step multi-fragment Gibson cloning. Genes of interest were obtained via custom gene synthesis from Comate Bioscience Co., Ltd. (Changchun, P.R. China). Alternatively, plasmids containing the desired genes can be purchased from Miaoling Plasmid Sharing Platform if applicable.

Transfection
Transient transfection was typically performed using Lipofectamine 3000 reagent (Cat# L3000001, ThermoFisher Scientific) in an 8-well (Cat# 155409) or 4-well (Cat# 155382) Lab-Tek®II imaging chamber from Thermo Scientific. In separate tubes, 0.375 µL of Lipofectamine 3000 reagent and 0.25 µg of DNA were each diluted in 12.5 µL Gibco™ Opti-MEM™ reduced-serum medium; then 0.5 µL P3000™ reagent was added to the diluted DNA. The diluted DNA with P3000 reagent was added to the diluted Lipofectamine 3000 reagent and incubated at room temperature for 5 minutes. Then 25 µL of the resulting complex was added into an imaging chamber well freshly seeded with 1.5-2.0×10^4 cells in 225 µL complete DMEM. The cells were maintained under 5% CO_2 at 37 ºC for around 2 h to allow cell adhesion. Afterwards, the medium was replaced by warm full DMEM and the cells were further incubated under 5% CO_2 at 37 ºC for over 20 h. For co-transfection of more than one plasmid, the quantity of DNA used in this protocol implies the total amount of plasmids.

Confocal microscopy
Live cells were imaged in phenol red free Dulbecco's Modified Eagle Medium (Cat# 21063-29, Life Technologies) supplemented with additional 10 % FBS, 1 % sodium pyruvate, 1 % NEAA, 1 % penicillin-streptomycin and 15 mM HEPES-Na (final pH 7.0) at 37 ºC under 5 % CO_2. Microscopy was performed using Zeiss LSM 880 inverted confocal laser scanning microscope equipped with an Airyscan super resolution module. Zeiss Plan-APOCHROMAT 63×/1.4 oil DIC objective was primarily used for imaging while Zeiss Plan-APOCHROMAT 100×/1.4 oil DIC objective was used as an alternative. Confocal images were typically acquired in 12-bit depth at 512×512 resolution. Typically, 488 nm argon laser was used to excite EGFP and HeNe laser 543 nm or HeNe laser 594 nm was used to excite mCherry. Other laser lines, e.g. 405 nm laser diode and 633 nm HeNe laser may also be used to excide appropriate fluorophores. In most cases, the basic imaging setup parameters were configured applying the *Smart Setup* function with typical parameters set as follows: scan speed 8, pixel dwell time 1.54 µs, number of averaging 4, line mode in one-direction scanning, and pinhole 89.9 µm. Where appropriate, edges of live cells recorded in microscopy were sketched as white lines for better clarity in this study.

Photoactivation
Smart Setup function was used to configure the basic parameters. Briefly, three default fluorophores should be selected including DAPI (to enable 405 nm laser passing through during photoactivation), EGFP (for imaging EGFP) and DsRed (for imaging mCherry). Then a critical step is to apply *Smartest* selection among three options. Three channels will thus be distributed into two tracks, one being EGFP and another being DAPI and DsRed, and both tracks will end up with sharing the same dichroic mirror configuration (MBS 488/543 and MBS 405). Under this configuration, 405 nm laser light can pass hence enabling photoactivation, Ar 488 nm laser line can be used to excite EGFP and HeNe543 nm laser line can be used to excite mCherry. For taking photoactivation time series, three functions including "Time Series", "Bleaching", and "Regions" should be activated in the *Acquisition* interface and the following will be more detailed parameters and settings. In *Acquisition Mode* panel, it was set *Scan Mode* as Frame, *Frame Size* as 512×512, *Speed* as 1.54 µs per pixel dwell, *Averaging* as 4× in 12-Bit, *Zoom Factor* around 2. In *Bleaching* panel, 405 nm laser was selected and the laser power was set as 50%. In *Time Series* panel, total 20 or 30 frames were set, photoactivation was triggered after the 5th frame and the bleaching will be repeated for 4 times. In *Regions* panel, a region such as in square shape should be selected which covers a whole cell or a local region of a cell and this region will be the region of photoactivation (ROP). After all these parameters being properly set, a photoactivation time series can be readily recorded by clicking the *Start Experiment* icon. For all photoactivation experiments, Zeiss Plan-APOCHROMAT 63×/1.4 oil DIC objective was used.

Preparation of the cyclic cell-penetrating peptide Cys-cR$_{10}$*
The cyclic decaarginine, HN_2-C(G)$_5$-cyclic(KrRrRrRrRrRE)-$CONH_2$, or Cys-cR$_{10}$*, composed of a cyclic rR ring (r = D-Arg, R = L-Arg) plus a (Gly)$_5$ linker with a N-terminal free cysteine. The peptide was synthesized via standard solid phase peptide synthesis using Rink amide resin. After the synthesis of liner R$_{10}$* fragment, intramolecular cyclization was performed to bridge the Lys side chain (-

NH_2 group) and Glu side chain (-COOH group). Afterwards, Cys-$(Gly)_5$ tail was sequentially added to the cyclic-R_{10}* moiety followed by TFA deprotection and HPLC purification. Cys-cR_{10}* peptide was obtained in a purity of over 95% and confirmed using mass spectrometry. $C_{84}H_{160}N_{50}O_{19}S$, exact mass: 2205.28, M.W.: 2206.56; found *m/z* 1103.45 $[M+2H]^{2+}$, *m/z* 442.20 $[M+5H]^{5+}$.

Föster resonance energy transfer (FRET)
EGFP and mCherry are a FRET pair because the spectra overlap between the emission spectrum of EGFP and the absorption spectrum of mCherry. In order to determine if energy transfer occurred from EGFP to mCherry-eDHFR with light illumination, 0.5 μM EGFP (1.0 equiv.), 0.5 μM mCherry-eDHFR (1.0 equiv.) were mixed in PBS buffer (pH 7.4) with or without adding 0.75 μM GBP-TMP(Nvoc) (1.5 equiv.). Light illumination was performed by irradiation of the reaction mixture with a 365 nm UV lamp (8 W) on ice for 5 min. Then, the fluorescence spectra of each reaction mixture (20 μl) before and after UV irradiation were recorded with a black 96-well plate using a microplate reader (Molecular Devices, SpectraMax iD3). Fluorescence spectra were recorded from 490-750 nm with excitation set at 470 nm.

UV-Vis absorption analysis
Trimethoprim (TMP), Nvoc-OH, and Cys-TMP(Nvoc) were first prepared as stock solutions in DMSO and then diluted in PBS buffer (pH 7.4) at 100 μM concentration (final 0.5%v of DMSO). 200 μl of these clear solutions were added into each well of a 96-well plate (Cat# 12599, Labselect) with a UV-transparent bottom (bottom surface 0.35 cm^2, solution column height 6 mm). The UV-Vis absorption spectra were recorded from 230 nm to 800 nm with 1 nm interval using a microplate reader (Molecular Devices, SpectraMax iD3). PBS buffer containing 0.5 %v of DMSO were used as the blank solution for subtraction of background absorption.

Pull-down and WB analysis
mCherry antibody (Cat #: bs-41161R, BIOSS) coated Protein A/G magnetic beads (Cat #: 36417ES03, Yeasen) were first prepared in advance according to manufacturer's protocol. In parallel, a mixture of EGFP (0.5 μM), mCherry-eDHFR (0.5 μM), and GBP-TMP(Nvoc) (0.75 μM) in PBS buffer (pH 7.4) were prepared and spitted to several vials (50 μl each). These vails were subjected to 0, 0.25, 0.5, 1, 3, 5, or 10 min 365 nm UV irradiation (8 W) on ice (for control group: no GBP-TMP(Nvoc) was added but 10 min of UV irradiation was applied). Following the manufacturer's protocol, these reaction solutions were mixed with mCherry antibody coated beads, slowly rotated at room temperature for 1 h, removal of supernatant with the help of a magnetic particle concentrator (MPC), washed by 1×TBST for two times, and removal of the supernatant again. The beads were mixed with 40 μl 2×SDS-PAGE loading buffer, boiled at 90 °C for 5 min, separated via SDS-PAGE (180 V, 50 min) using 12 % gel, transferring the proteins to nitrocellulose membrane (Cat #: 66485, PALL) using a rapid transferring buffer (Cat #: GF1816, Genefist). The protein transferred membrane was subsequently subjected to Western blot analysis using EGFP rabbit monoclonal antibody (Cat #: R24437, Zenbio, 1:1000) and mCherry mouse monoclonal antibody (Cat #: bs-33131M, BIOSS, 1:1000) as the primary antibody, and HRP-conjugated goat anti-rabbit IgG antibody (Cat #: 511203, Zenbio) or HRP-conjugated goat anti-mouse IgG antibody (Cat #: 511103, Zenbio) was used as the secondary antibody.

Protein expression and purification
pTXB1 vector was used to express intein-tag fused nanobody chimeras for expressed protein ligation (EPL). The plasmids for protein expression were first transformed into *E. coli* Rosetta 2a cells and the transformants were selected on ampicillin (125 mg·L^{-1}) agar plates depending on the antibiotic resistance of the plasmids. A single colony was used to inoculate 50-100 ml of LB medium containing 100 mg·L^{-1} ampicillin and shaken at 240 rpm for 8-10 hours or overnight at 37 °C. 30-50 ml of the preculture was used to further inoculate ~1.8 L fresh LB medium containing 125 mg·L^{-1} ampicillin and additional chloramphenicol (33 mg·L^{-1}). The absorbance at 600 nm (OD 600) of the inoculated culture should be controlled between 0.05 to 0.1 in this inoculation step. Then the culture was shaken at 180 rpm at 37°C for a few hours (typically 2-3 h) until OD 600 reached 0.5-0.6. Then 0.5 ml isopropyl β-D-thiogalactoside (IPTG) stock solution (1M) was added (final ~0.27 mM) to induce protein expression at 16 °C overnight.

Later, cells were harvested by centrifugation (8000 rpm, 4 °C, 15 min) and washed once with PBS (4700 rpm, 10 min). The bacterial pellet was resuspended in lysis buffer (pH 8.0, PBS supplemented with additional 0.5 M NaCl, 3 % glycerol, 3 mM β-mercaptoethanol (BME), 1 mM phenylmethylsulfonyl fluoride (PMSF). Cells were typically lysed using ultra-high-pressure homogenizer cooled by a bench chiller for 2-3 cycles under 800-900 bar at 4 °C. The lysate was cleared by high-speed centrifugation (22500 rpm, 45 min, 4 °C) and the supernatant was loaded onto a gravity Ni-NTA column (2-5 ml resin). The Ni-NTA column was washed and then the His-tag fused protein was eluted using step-gradient of imidazole (50, 100, …, until 500 mM) in elution buffer (pH 8.0 PBS, supplemented with additional 0.5 M NaCl, 3 % glycerol and 3 mM BME). The obtained proteins were typically concentrated and buffer exchanged in elution buffer without 0.75 mM BME. Then the proteins were aliquoted, snap frozen in liquid nitrogen, and stored under -80 °C.

Stepwise protocol for assembly of PANCIDs:

- EPL: 0.9 ml of nanobody-intein-His_6 chimera at around 20 mg·ml^{-1} concentration in a 2 ml Eppendorf tube was added with 0.45 ml MENSNa (pH 8.0, 2 M) and 0.45 ml MPAA (pH 8.0, 1.1 M) stock solutions. Afterwards, 22.5 μl of Cys-TMP(Nvoc) (60 mM/DMSO) stock solution was added at final concentration around 0.75 mM. The reaction solution was degassed via sonification, charged with argon, sealed, shielded from light using aluminum foil, and incubated at 4 °C for 3 days to finish EPL.
- Reverse Ni-IMAC purification: The reaction mixture was subjected to Ni-NTA column purification to separate nanobody-TMP(Nvoc) conjugate. Pure conjugate fractions were combined, buffer exchanged in BME-free elution buffer (pH 8.0 PBS, supplemented with additional 3%v glycerol and 0.5M NaCl), degassed via sonication, added with 2 equiv. TCEP (20 mM stock), charged with argon, incubated at 4 °C for 45 min. Subsequently, 8 equiv. of DTNB (100 mM) stock solution in 0.5 M Na_2HPO_4 buffer was added portion-wise (2 equiv. × 4). The reaction solution will immediately turn yellowish, and the reaction solution was incubated at 4 °C under argon for 60 min. **Abbreviation:** IMAC, immobilized metal affinity chromatography.
- Disulfidization coupling: Buffer exchange into pH 9.0 disulfidization buffer (50 mM HEPES, 0.5 M NaCl) for three times, degas via sonification, add around 3 equiv. of Cys-cR_{10}* (15 mM/ DMSO stock), degas via sonification, charged with argon, and incubate on ice overnight to finish the coupling. The next day, the reaction solution was buffer exchanged two times into PBS, analyzed via non-reducing SDS-PAGE, aliquoted, snap frozen in liquid nitrogen, and stored under -80 °C before use.

Image analysis and statistics

Microscopic images were analyzed and processed with ImageJ/Fiji and prepared for presentation using Microsoft Office PowerPoint. Image manipulations were restricted to adjustment of brightness level, background subtraction, cropping, rotating, scaling, and false color-coding using Look-Up Tables (LUT). All microscopic imaging experiments were representative of at least three repeats if not otherwise stated. No randomization nor blinding was used in this study. Origin and Microsoft Excel were used for plotting, data fitting, graphing and statistical analysis. Student's *t*-Tests were used to compare two experimental conditions. Paired *t*-Tests were performed if measurements were linked, as for example concerning pre- versus post-uncaging conditions. Otherwise, unpaired tests were used as for example cells with or without drug treatment. When necessary, stars were used to denote *P*-values for indicated statistical tests (*: $P<0.05$; **: $P<0.01$; ***: $P<0.001$; ****: $P<0.0001$). Exact *P*-values were indicated for critical experiments.

All box plots show mean (square), median (bisecting line), bounds of box (75th to 25th percentiles), outlier range with 1.5 coefficient (whiskers), and minimum and maximum data points (lower/ upper whiskers). Pearson's correlation coefficient (PCC) was employed for colocalization analysis using the “Manders_Coefficients.class” plugin for ImageJ/Fiji. Usually, there is only a single cell within an imaging field; otherwise, a single cell was first selected using the polygon selections tool and then remove extra cells (Edit/Clear outside) prior to PCC analysis. The images were converted to 8-bit depth (Image/Type/8-bit) prior to analysis.

Organic Synthesis

General

Unless otherwise specified, all chemicals were purchased from commercial vendors and were used without further purification. The ^{1}H- and ^{13}C-NMR spectra were measured on a 600 MHz Bruker BioSpin GmbH magnetic resonance spectrometer. Data for ^{1}H-NMR spectra are reported as follows: Chemical shifts are reported as δ in units of parts per million (ppm); multiplicities are reported as follows: s (singlet), d (doublet), t (triplet), q (quartet), dd (doublet of doublets), m (multiplet), or br (broadened); coupling constants are reported as *J* values in Hertz (Hz); the number of protons (n) for a given resonance is indicated as nH, and is based on the spectra integration values. High resolution mass spectra (HR-MS) measurement was performed via a customer service using electron spray ionization (ESI).

Synthesis of Cys-PEG8-TMP(Nvoc) (1) and related key intermediates

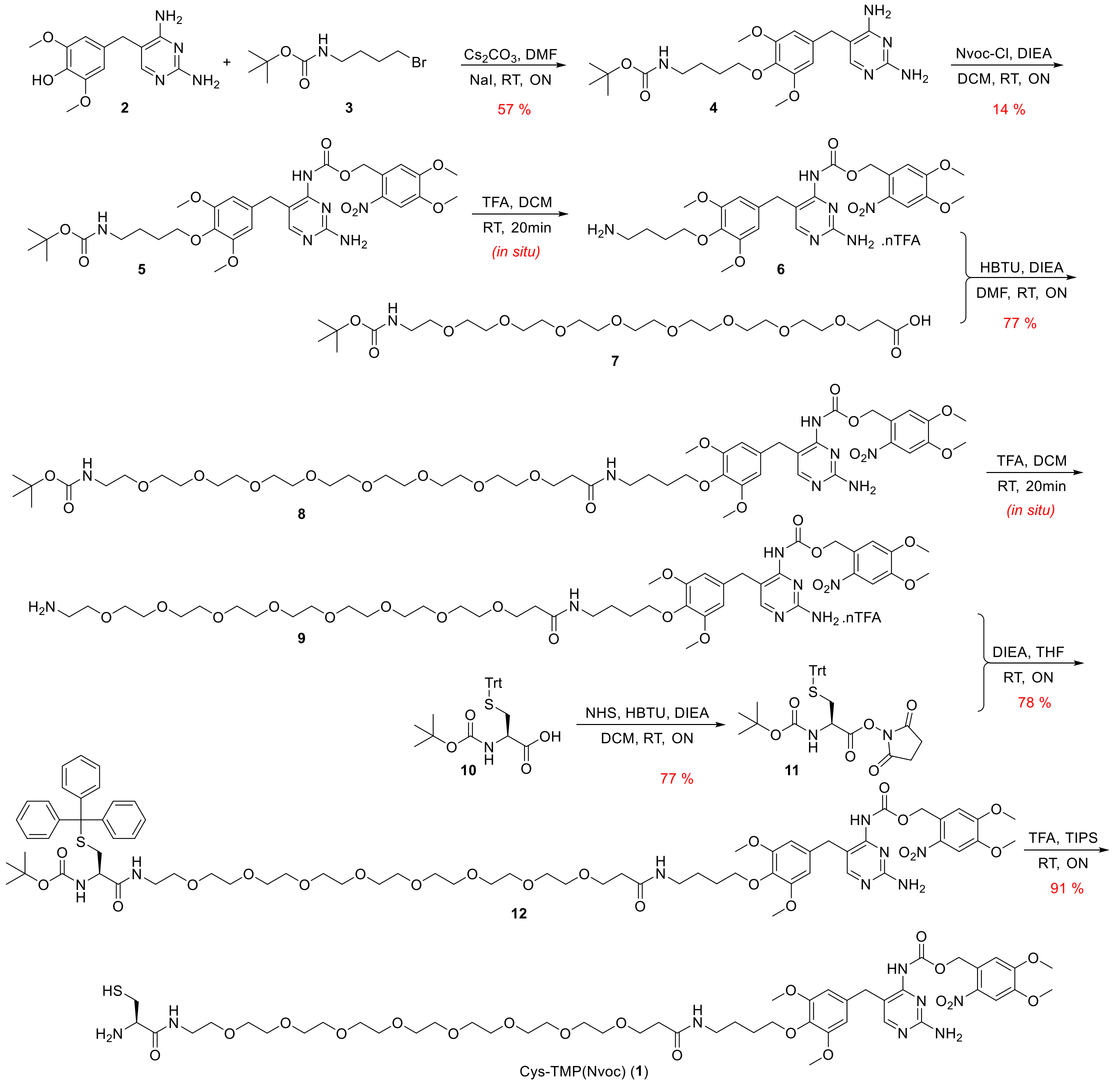

Cys-TMP(Nvoc) (**1**)

Scheme S1. Synthetic scheme toward Cys-TMP(Nvoc). Abbreviations: DIEA, *N*,*N*-diisopropylethylamine; RT, room temperature; ON, overnight; TFA, trifluoroacetic acid; DCM, dichloromethane; HBTU, 2-(1*H*-Benzotriazole-1-yl)-1,1,3,3-tetramethyluronium hexafluorophosphate; DMF, *N*,*N*-dimethylformamide; TIS, triisopropylsilane.

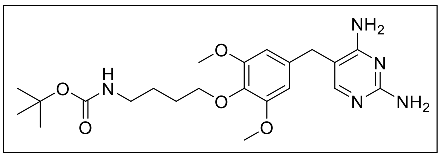

***tert*-Butyl (4-(4-((2,4-diaminopyrimidin-5-yl)methyl)-2,6-dimethoxyphenoxy)butyl)carbamate** (TMP-BuNHBoc, **4**). Dimethoprim, i.e. TMP-OH (440 mg, 1.44 mmol) and *tert*-butyl *N*-(4-bromobutyl)carbamate (383.4 mg, 1.52 mmol), NaI (216 mg, 1.44 mmol) and Cs_2CO_3 (985.8 mg, 3.02 mmol) were combined inside a thoroughly dried two neck round-bottom-flask (RBF) equipped with a stir bar

under Ar. Then anhydrous DMF (7.2 mL, 0.1 M) was injected and the reaction mixture were stirred at room temperature (RT) overnight to complete the coupling. DMF solvent was removed under reduced pressure (2 mbar, 50°C) and the residue was partitioned between EtOAc/Na_2CO_3 (aq.). The organic layer was separated and aqueous phase was extracted two additional times by EtOAc. All organic layers were combined, wash with small volumes of brine twice, dried over anhydrous Na_2SO_4, filtered, concentrated and purified via silica gel chromatography (DCM: MeOH 15:1) to give 370 mg light-yellow solid as the product in a yield of 57%. **^{1}H-NMR** (DMSO-d^6, 600MHz): *δ* 7.50 (s, 1H), 6.80 (t, *J*=5.88Hz, 2H), 6.54 (s, 2H), 6.15 (s, br, 2H), 5.75 (s, br, 2H), 3.76 (t, 2H, *J*=6.15), 3.70 (s, 6H), 3.51 (s, 2H), 2.94 (q, *J*=6.54Hz, 2H), 1.55 (m, 2H), 1.50 (m, 2H), 1.37 (s, 9H); **^{13}C-NMR** (DMSO-d^6, 151MHz): *δ* 162.27, 161.96, 155.62, 155.13, 152.87, 135.62, 134.8, 105.91, 105.82, 77.33, 72.04, 55.83, 39.58, 32.97, 28.30, 27.04, 26.13; **HRMS**(ESI): $C_{22}H_{34}N_5O_5^+$ [M+H]$^+$, calcd. 448.2560, found 448.2559.

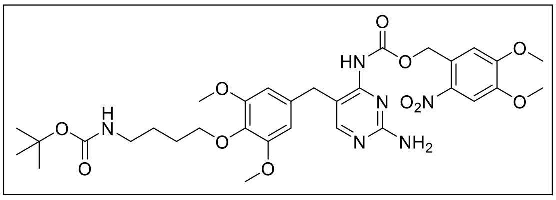

***tert*-butyl (4-(4-((2-amino-4-((((4,5-dimethoxy-2-nitrobenzyl)oxy)carbonyl)amino)pyrimidin-5-yl)methyl)-2,6-dimethoxyphenoxy)butyl)carbamate** (*N*^{4}-NvocTMP-BuNHBoc, **5**). TMP-BuNHBoc (370 mg, 0.83 mmol) from the last step and Nvoc-Cl (250.6 mg, 0.91 mmol) were added into a thoroughly dried 2 neck RBF equipped with a stir bar under Argon. Anhydrous DCM (8.3 ml, 0.1 M) was added to give a solution followed by addition of DIEA (1173 mg, 1.82 mmol). This reaction solution was allowed stirred at room temperature overnight. DCM was removed under reduced pressure and the residue was partitioned between EtOAc/Na_2CO_3 (aq.). The organic layer was separated and the aqueous phase was extracted two additional times by EtOAc. All organic phases were combined, washed with brine for two times, dried over anhydrous Na_2SO_4, filtered, concentrated, and purified via gradient silica gel chromatography (MeOH/DCM 1% to 3%) to give 78.4 mg of light-yellow solid as the product in a yield of 14%. **TLC** (DCM:MeOH 20:1): Rf 0.3; **^{1}H-NMR** (DMSO-d^6, 600MHz): *δ* 9.78 (s, 1H), 8.03 (s, 1H), 7.72 (s, 1H), 7.22 (s, 1H), 6.79 (t, *J*=5.76Hz, 1H), 6.47 (s, 1H), 6.41 (s, 1H), 5.41 (s, 2H), 3.87 (s, 3H), 3.84 (s, 3H), 3.72 (s, 2H), 3.70 (t, *J*=6.48Hz, 2H), 3.64 (s, 6H), 2.93 (q, *J*=6.42Hz, 2H), 1.53 (m, 2H), 1.47 (m, 2H), 1.36 (s, 9H); **^{13}C-NMR** (DMSO-d^6, 600MHz): *δ* 170.38, 162.47, 160.20, 156.52, 155.61, 153.38, 152.89, 152.68, 147.87, 139.33, 135.54, 134.80, 126.98, 114.76, 110.84, 108.19, 105.62, 77.32, 71.99, 63.22, 59.79, 56.22, 56.12, 55.67, 39.58, 33.36, 28.29, 27.03, 26.11, 20.80, 14.11; **HRMS**(ESI): $C_{32}H_{43}N_6O_{11}^+$ [M+H]$^+$, calcd. 687.2990, found 687.2990.

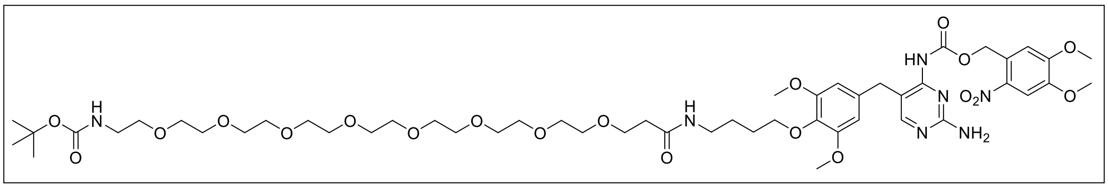

4,5-dimethoxy-2-nitrobenzyl (2-amino-5-(4-((2,2-dimethyl-4,32-dioxo-3,8,11,14,17,20,23,26,29-nonaoxa-5,33-diazaheptatriacontan-37-yl)oxy)-3,5-dimethoxybenzyl)pyrimidin-4-yl)carbamate (*N*^{4}-NvocTMP-PEG$_8$-NBoc, **8**). First of all, *N*^{4}-NvocTMP-BuNHBoc was subjected to deprotection *in situ* to remove the Boc protecting group and liberate the primary amino group for subsequent amide coupling. Briefly, *N*^{4}-NvocTMP-BuNHBoc (95.3 mg, 0.14 mmol) was dissolved in anhydrous DCM (2 ml) inside a thoroughly dried RBF equipped with a stirrer bar under argon. Then TFA (1 ml) was added dropwise and the solution was stirred under argon for 20 minutes to allow complete deprotection. DCM and TFA were removed under reduced pressure and then dried *in vacuo* to give a residue as the crude intermediate. Small volumes of MeOH were added to dissolve the residue. MeOH was first removed under reduced pressure and then the residue was further dried under high vacuum for a short while. This process was helpful to remove residual TFA inside the deprotected product. This process was repeated two additional times to remove most residual TFA. 122.5 mg of yellowish solid was obtained as the amine intermediate in the form of TFA salt (it can be calculated that n=2.6 for TFA in the salt formula). Afterwards, *N*^{4}-NvocTMP-BuNH$_2$.nTFA (122 mg, 0.14 mmol), BocNH-PEG$_8$-COOH (118.7 mg, 0.219 mmol) and HBTU (87.2 mg, 0.23 mmol) were combined inside a dried 2 neck RBF equipped with a stirrer bar. Anhydrous DMF (2.1 ml, 0.1 M) was added followed by the addition of DIEA (107.8 mg, 0.836 mmol). The reaction mixture was stirred at room temperature overnight to complete the coupling. DMF was removed under reduced pressure (2 mbar, 50 °C), and the residue was partitioned between EtOAc/Na_2CO_3 (aq.). The organic layer was separated and the aqueous phase was extracted two additional times by EtOAc. All organic layers were combined, washed twice with brine, dried over anhydrous Na_2SO_4, filtered, concentrated and purified via silica gel chromatography via step gradient elution (DCM:MeOH 30:1 → DCM:MeOH 20:1) to give 119 mg yellowish oil as the product in a yield of 77%. **TLC** (DCM:MeOH 15:1): Rf 0.3; **^{1}H-NMR** (DMSO-d^6, 600MHz): *δ* 9.78 (s, 1H), 8.02 (s, 1H), 7.81 (t, *J*=5.76Hz, 1H), 7.72 (s, 1H), 7.22 (s, 1H), 6.76 (t, *J*=5.76Hz, 1H), 6.48 (s, 2H), 6.42 (s, 2H), 5.76 (s, 2H), 5.41 (s, 2H), 3.87 (s, 3H), 3.84 (s, 3H), 3.71 (s, 2H), 3.70 (t, *J*=6.3Hz, 2H), 3.64 (s, 6H), 3.58 (t, *J*=6.96Hz, 2H), 3.45-3.52 (m, 28H), 3.36 (t, *J*=6.18Hz, 2H), 3.05 (t, m, 4H), 2.29 (t, *J*=6.0Hz, 2H), 1.55 (m, 2H), 1.51 (m, 2H), 1.36 (s, 9H); **^{13}C-NMR** (DMDO-d^6, 151MHz): *δ* 169.85, 162.42, 160.11, 156.56, 155.60, 153.39, 152.91, 152.67, 147.88, 139.35, 135.54, 134.81, 126.97, 114.74, 110.87, 108.19, 105.63, 77.60, 71.91, 69.79, 69.74, 69.69, 69.54, 69.52, 69.18, 66.92, 63.23, 56.22, 56.13, 55.68, 38.18, 36.18, 33.35, 28.24, 27.11, 25.69; **HRMS**(ESI): $C_{51}H_{80}N_7O_{20}^+$ [M+H]$^+$, calcd. 1110.5458, found 1110.5455.

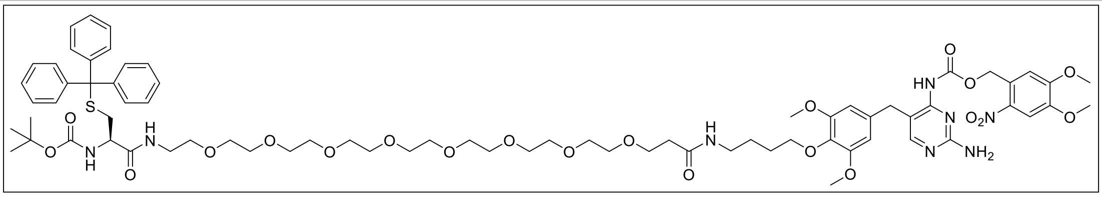

4,5-Dimethoxy-2-nitrobenzyl (R)-(2-amino-5-(4-((2,2-dimethyl-4,7,35-trioxo-6-((tritylthio)methyl)-3,11,14,17,20,23,26,29,32-nonaoxa-5,8,36-triazatetracontan-40-yl)oxy)-3,5-dimethoxybenzyl)pyrimidin-4-yl)carbamate (BocCys(Trt)-PEG$_8$-TMP(Nvoc), **12**). The reagent BocCys(Trt)OSu was prepared from BocCys(Trt)OH by coupling with NHS using HBTU. In parallel, *N*^{4}-NvocTMP-PEG$_8$-NHBoc was deprotected to remove the Boc protective group to liberate the free primary amine. Briefly, *N*^{4}-NvocTMP-PEG$_8$-NHBoc (110.2 mg, 0.1 mmol) was dissolved in anhydrous DCM (2 ml) and then TFA (1 ml) was added. The reaction solution was stirred at room temperature under argon for 20 min to complete the deprotection. Afterwards, DCM and TFA were removed under reduced pressure and then dried *in vacuo*. Subsequently, a small amount of MeOH was added to dissolve the residue. MeOH was removed under reduced pressure and the residue was dried *in vacuo*. This MeOH addition/vacuum drying process was repeated for two additional times to get ride of residual TFA inside the deprotected amine intermediate. After dried *in vacuo*, total 116.2 mg of light oil intermediate *N*^{4}-NvocTMP-PEG$_8$-NH$_2$·nTFA was obtained in the form of TFA salt (n=1.4 for TFA as calculated). Next, *N*^{4}-NvocTMP-PEG$_8$-NH$_2$·nTFA (50 mg, 0.039 mmol) and BocCys(Trt)-OSu (27.5 mg, 0.49 mmol) were added into a two neck RBF equipped with a stirrer bar under argon. Anhydrous THF (0.86 ml) was added followed by the addition of DIEA (22.2 mg, 0.172 mmol). The reaction mixture was stirred at room temperature overnight to complete the coupling. THF was removed under reduced pressure and the residue was partitioned between EtOAc/Na_2CO_3 (aq.). The organic layer was separated and the aqueous phase was extracted two additional times by EtOAc. All organic layers were combined, washed two times by brine, dried over anhydrous Na_2SO_4, filtered, concentrated and purified via silica gel chromatography (EtOAc:MeOH 5:1) to give 38.2 mg light yellow solid as the product in a yield of 67%. **TLC** (EtOAc:MeOH 5:1): Rf ~0.3; **^{1}H-NMR** (DMSO-d^6, 600MHz): *δ* 9.78 (s, 1H), 8.02 (s, 1H), 7.81 (t, *J*=6.84Hz, 1H), 7.76 (t, *J*=6.36Hz, 1H), 7.72 (s, 1H), 7.35-7.30 (m, 6H), 7.29-7.26 (m, 6H), 7.26-7.21 (m, 4H), 6.90 (d, *J*=8.58Hz, 1H), 6.49 (s, 2H), 6.41 (s, 2H), 5.41 (s, 2H), 3.92 (t, *J*=7.98, 1H), 3.89 (s, 3H), 3.84 (s, 3H), 3.72 (s, 2H), 3.71 (m, 2H), 3.64 (s, 6H), 3.57 (t, *J*=6.42Hz, 2H), 3.52-3.42 (m, 32H), 3.18 (m, 1H), 3.11 (m, 1H), 3.06 (m, 2H), 3.28 (m, 2H), 1.58-1.49 (m, 4H), 1.36 (s, 9H); **^{13}C-NMR** (DMDO-d^6, 151MHz): *δ* 170.12, 169.85, 162.47, 160.19, 156.53, 154.91, 153.39, 152.91, 152.89, 147.88, 144.34, 139.34, 135.56, 134.81, 129.11, 128.07, 126.98, 126.78, 114.76, 110.86, 108.19, 105.63, 78.37, 71.92, 69.79, 69.74, 69.69, 69.63, 69.55, 68.89, 66.92, 65.86, 63.23, 59.80, 56.23, 56.13, 55.68, 53.39, 38.68, 38.19, 36.18, 34.06, 33.36, 28.15, 27.12, 25.69; **HRMS**(ESI): $C_{73}H_{99}N_8O_{21}S^+$ [M+H]$^+$, calcd. 1455.6645, found 1455.6636.

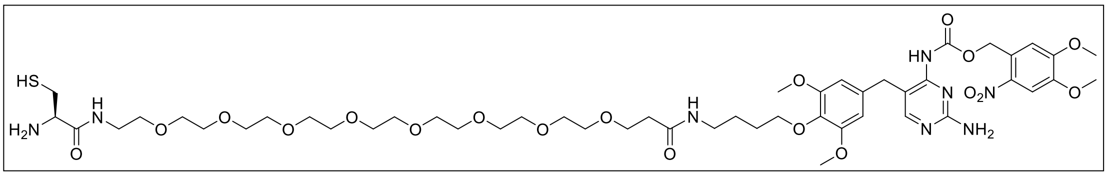

4,5-Dimethoxy-2-nitrobenzyl (R)-(2-amino-5-(4-((2-amino-1-mercapto-3,31-dioxo-7,10,13,16,19,22,25,28-octaoxa-4,32-diazahexatriacontan-36-yl)oxy)-3,5-dimethoxybenzyl)pyrimidin-4-yl)carbamate (Cys-TMP(Nvoc), **1**). For the deprotection, BocCys(Trt)-PEG$_8$-TMP(Nvoc) (26 mg, 0.018 mmol) from the last step was dissolved in TFA (2 ml) and then 2.5%V triisopropylsilane (50 μl) was added. The reaction mixture was stirred at room temperature under argon for 1.5 h. Then, volatile components were removed under reduced pressure and the residue was dried *in vacuo*. The residue was subjected to MeOH addition/vacuum drying process for three times as mentioned previously to remove any residual volatile components such as TFA from the deprotected product. Afterwards, the dried residue was partitioned between EtOAc/ddH$_2$O. The aqueous layer was separated and washed two additional times by EtOAc to give 16.2 mg of light yellowish wax-like solid as the final product (n=1.5 for TFA) in a yield of 71%. **^{1}H-NMR** (DMSO-d^6, 600MHz): *δ* 10.17 (s, 1H), 8.57 (t, *J*=5.64Hz, 1H), 8.27 (br, 3H, -NH_3^+), 8.01 (s, 1H), 7.83 (m, 1H), 7.73 (s, 1H), 7.51 (br, 2H), 7.26 (s, 1H), 6.61 (s, 0.25×2H), 6.48 (s, 0.75×2H), 5.46 (s, 2H), 3.95 (t, *J*=5.34Hz, 1H), 3.88 (s, 3H), 3.86 (s, 3H), 3.78 (br, 2H), 3.73 (m, 2H), 3.72 (s, 2H), 3.66 (s, 6H), 3.58 (t, *J*=6.54Hz, 2H), 3.52-3.44 (m, 30H), 3.36 (m, 1H), 3.25 (m, 1H), 3.07 (m, 2H), 2.91 (br, 1H), 2.29 (t, *J*=6.48Hz, 2H), 1.56 (m, 2H), 1.53 (m, 2H); **^{13}C-NMR** (DMSO-d^6, 151Hz): δ 169.91, 166.79, 164.08, 154.39, 153.35, 153.09, 153.02, 151.67, 148.09, 139.63, 135.05, 134.22, 132.89, 126.28, 113.90, 111.54, 108.94, 108.26, 106.24, 105.89, 71.95, 69.80, 69.75, 69.70, 69.63, 69.55, 68.76, 66.93, 63.77, 56.31, 56.17, 55.93, 55.76, 53.93, 38.94, 38.19, 36.18, 32.68, 32.13, 30.49, 27.13, 25.71, 25.20, 24.31; **HRMS**(ESI): $C_{49}H_{77}N_8O_{19}S^+$ [M+H]$^+$, calcd. 1113.5026, found 1113.5034; **HRMS**(ESI): $C_{49}H_{77}N_8O_{19}S^+$ [M+H]$^+$, calcd. 1113.5026, found 1113.5034.

NMR Spectra

TMP-BuNHBoc: **^{1}H-NMR** (600MHz, DMSO-d^{6})

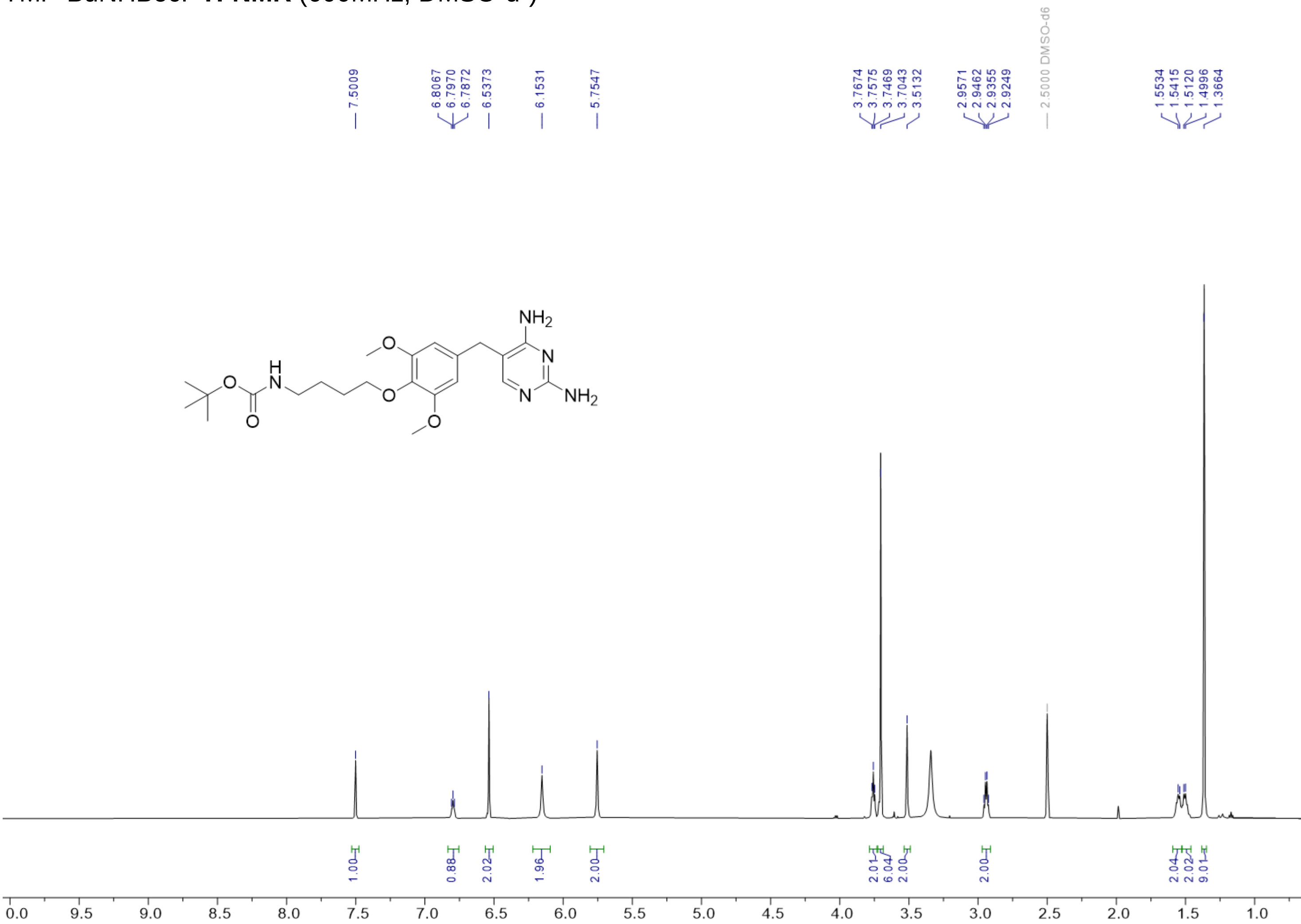

TMP-BuNHBoc: **^{13}C-NMR** (600MHz, DMSO-d^{6})

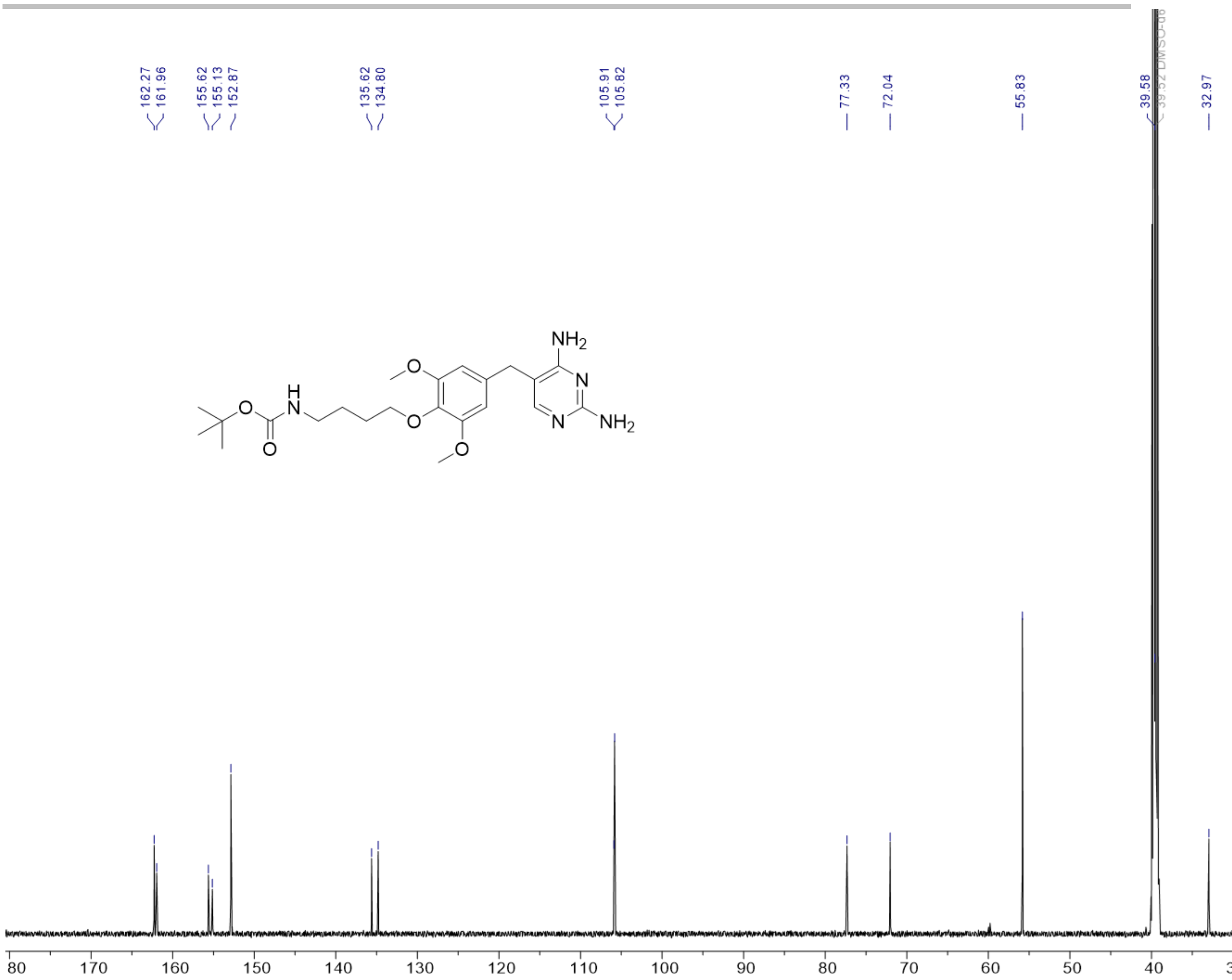

*N*4-NvocTMP-BuNHBoc: **1H-NMR** (600MHz, DMSO-d6)

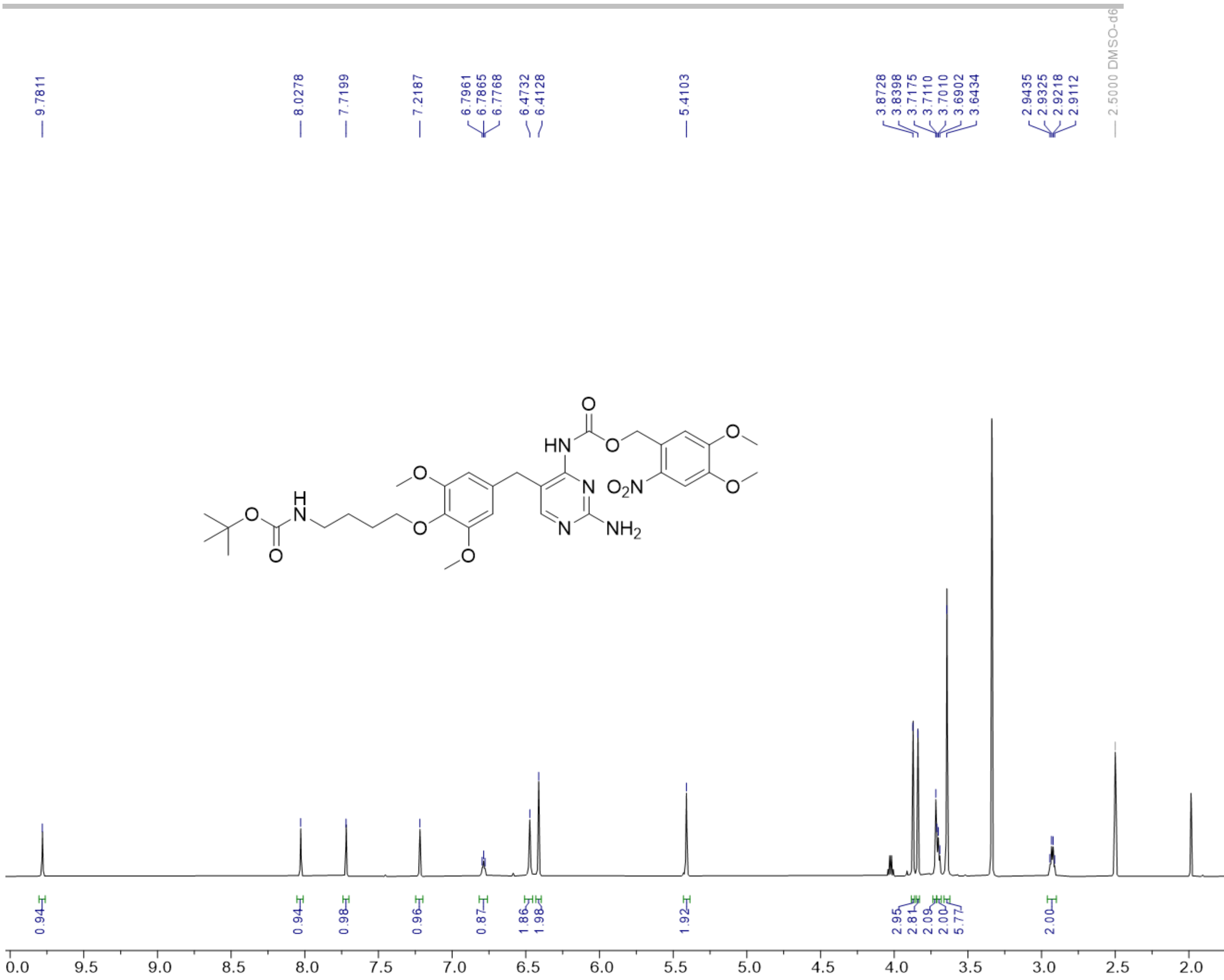

N[4]-NvocTMP-BuNHBoc: **^{13}C-NMR** (600MHz, DMSO-d[6])

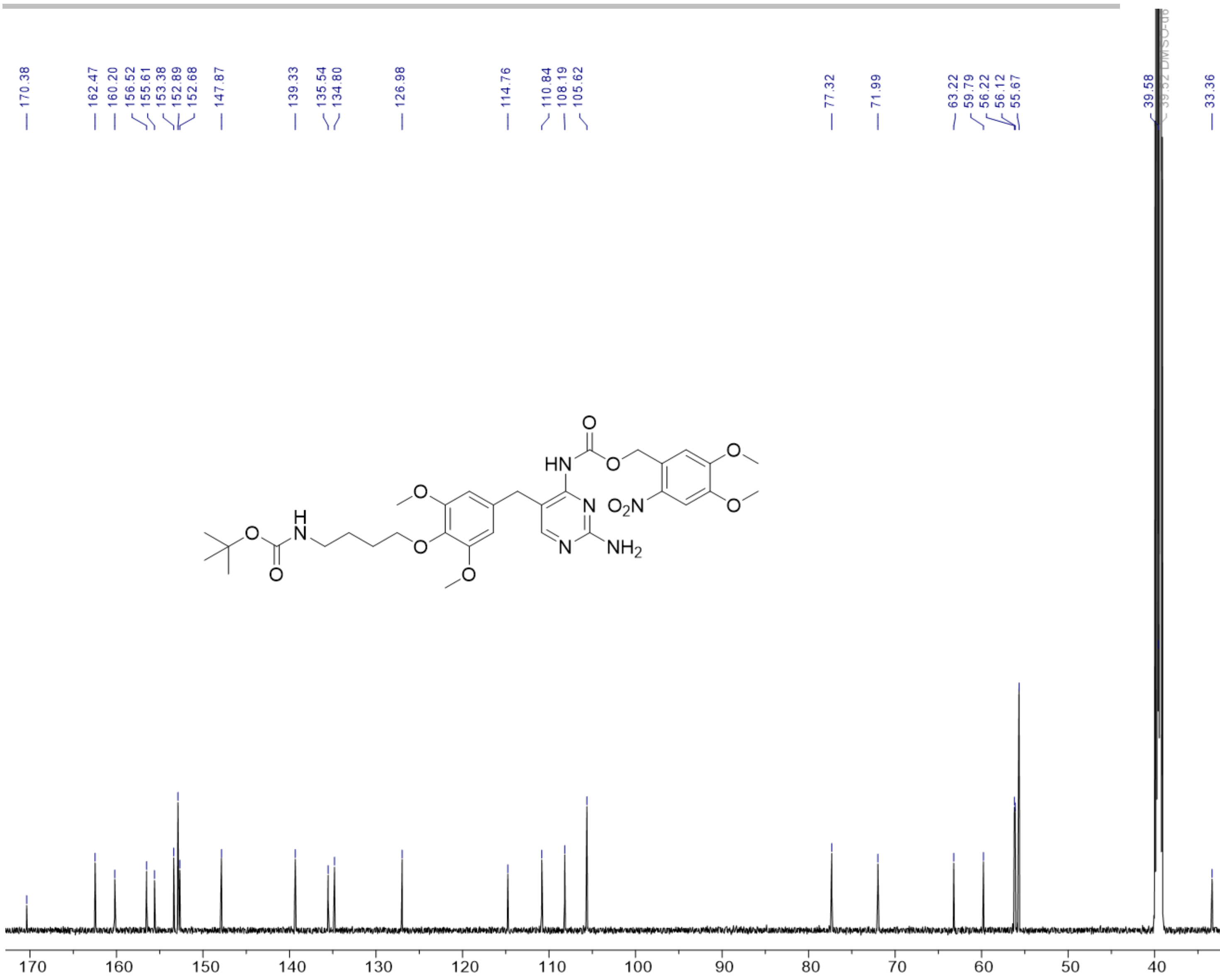

N[4]-NvocTMP-BuNHBoc: **HSQC** (600MHz, DMSO-d[6])

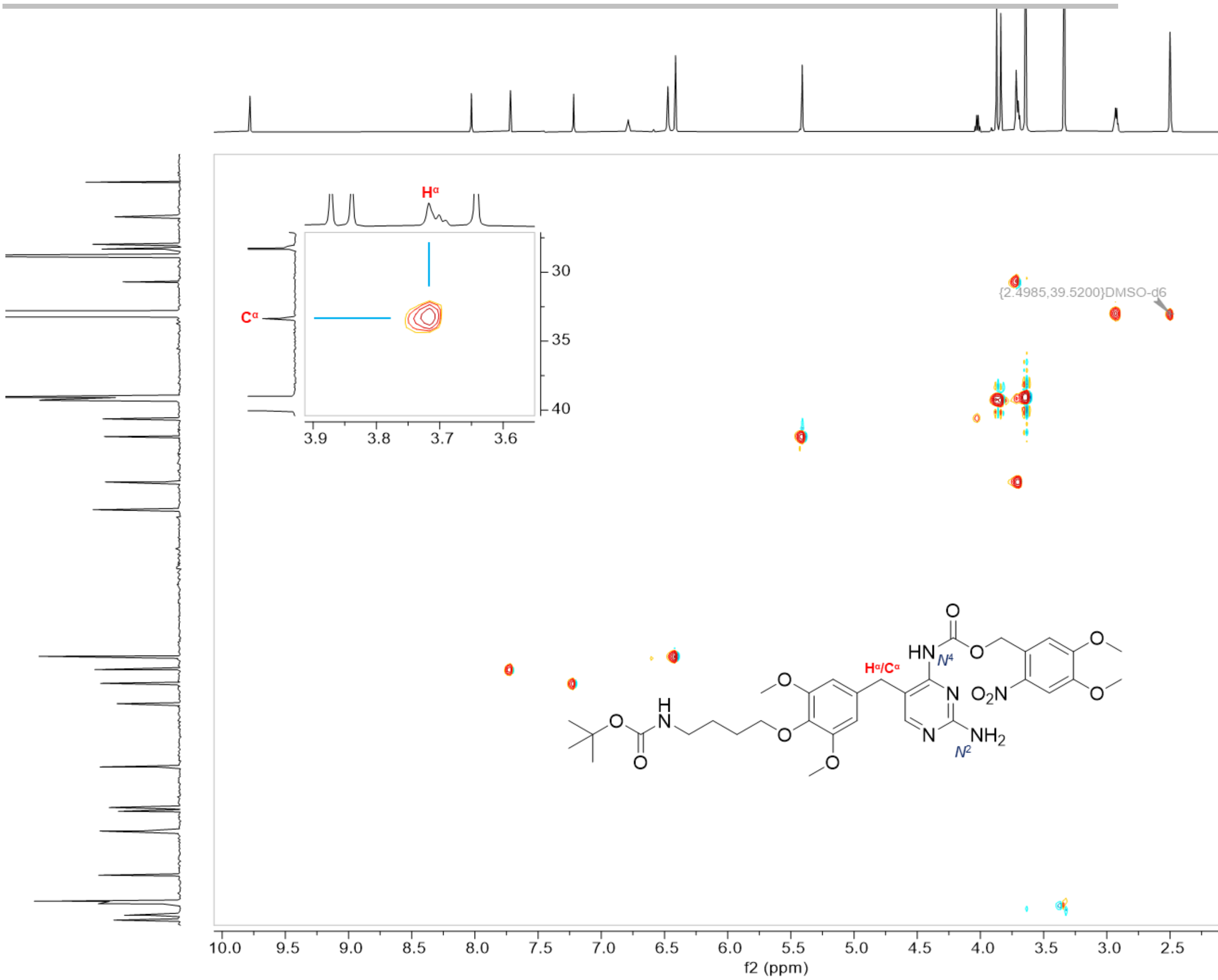

*N*4-NvocTMP-BuNHBoc: **NOESY** (600MHz, DMSO-d6)

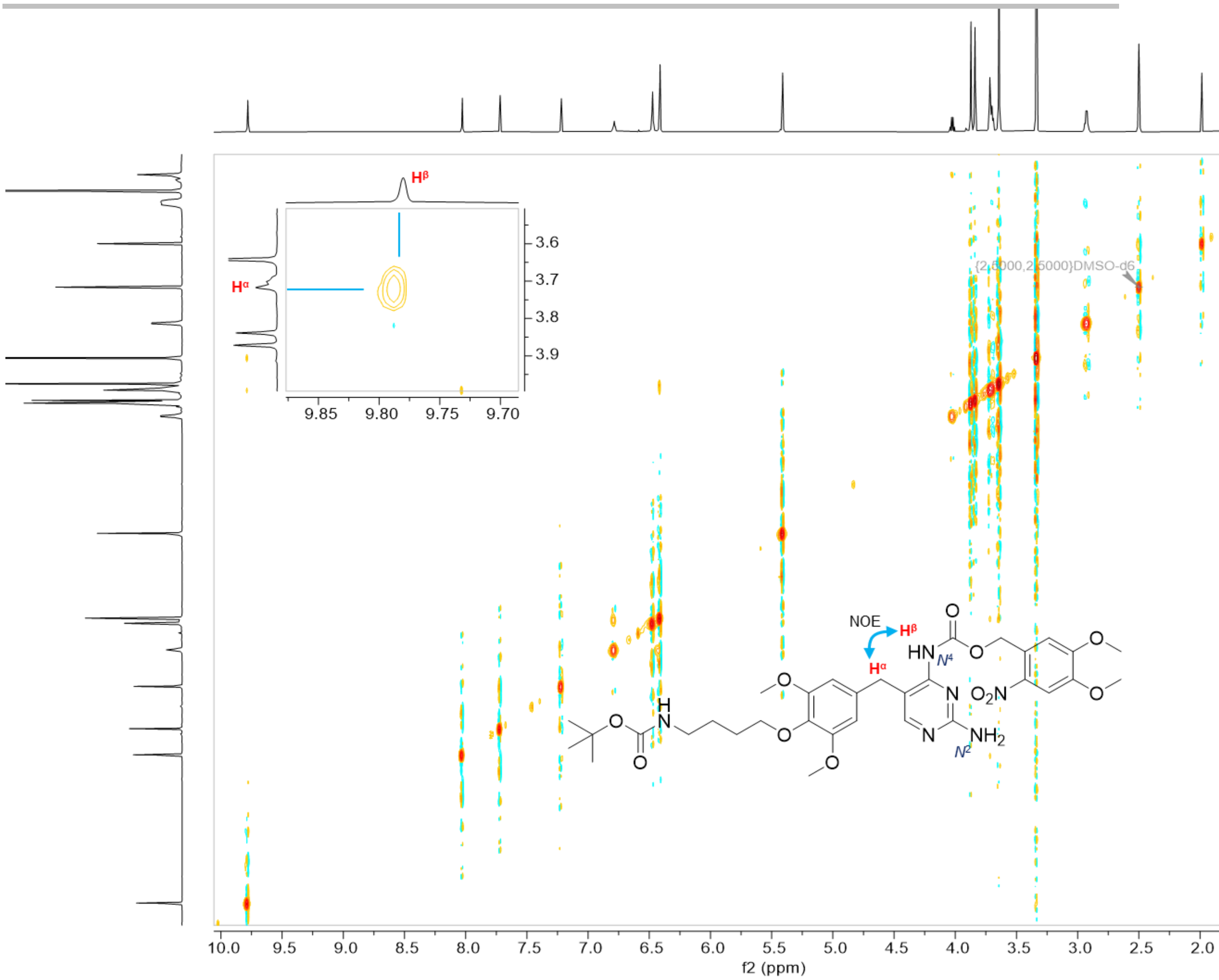

N[4]-NvocTMP-PEG$_8$-NHBoc: **^{1}H-NMR** (600MHz, DMSO-d^6)

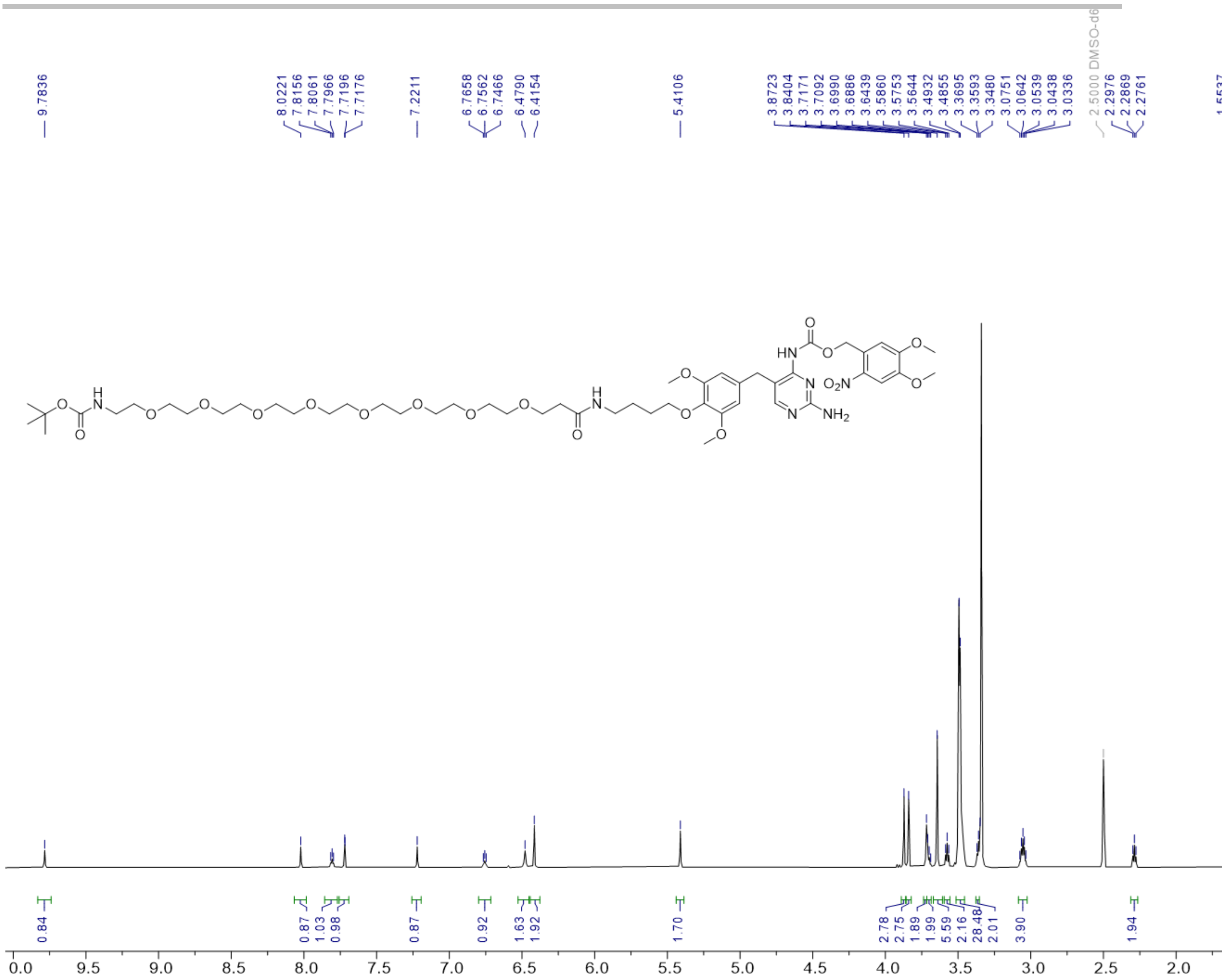

N^4-NvocTMP-PEG$_8$-NHBoc: **^{13}C-NMR** (600MHz, DMSO-d^6)

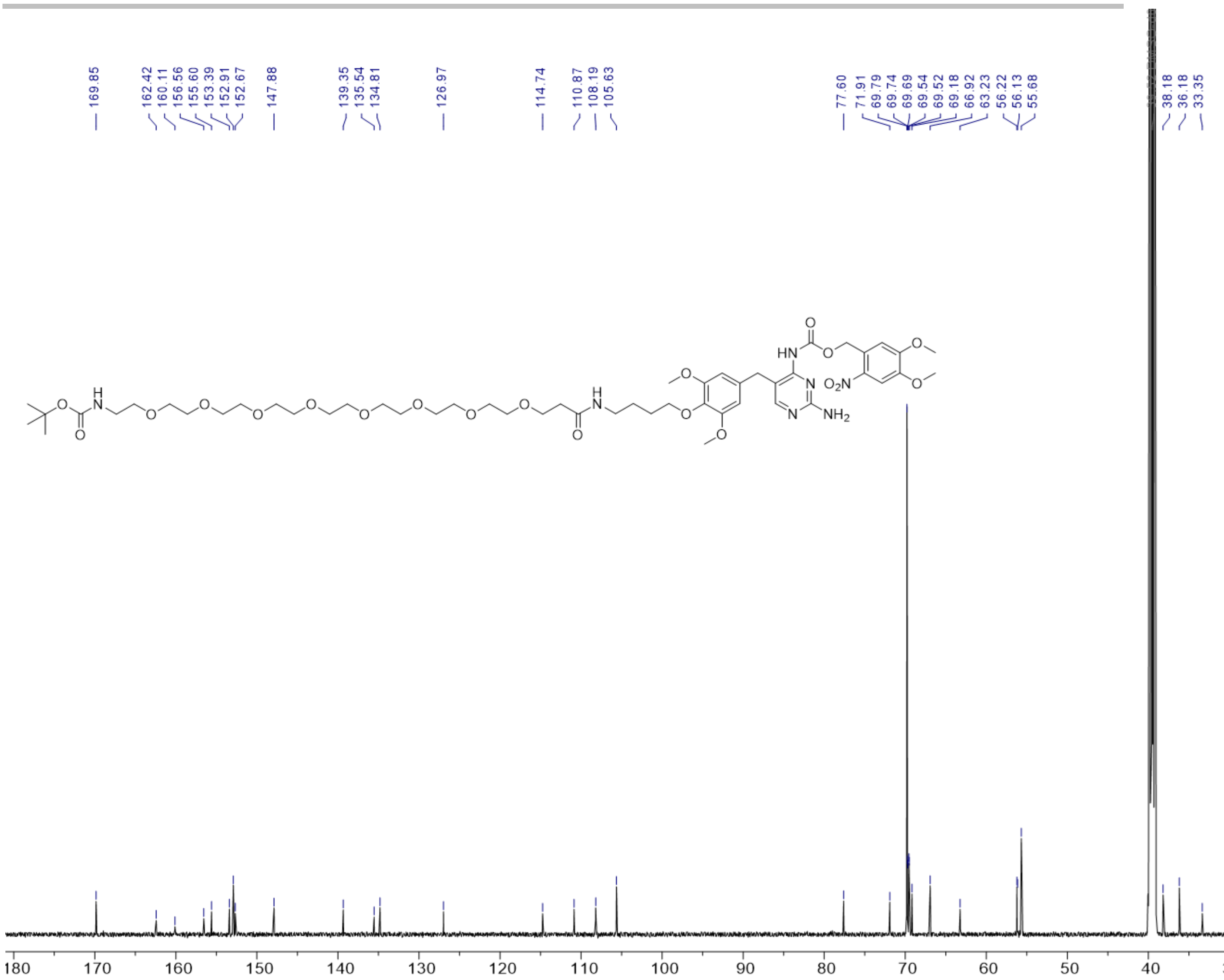

BocCys(Trt)-PEG$_8$-TMP(Novc): **^{1}H-NMR** (600MHz, DMSO-d^6)

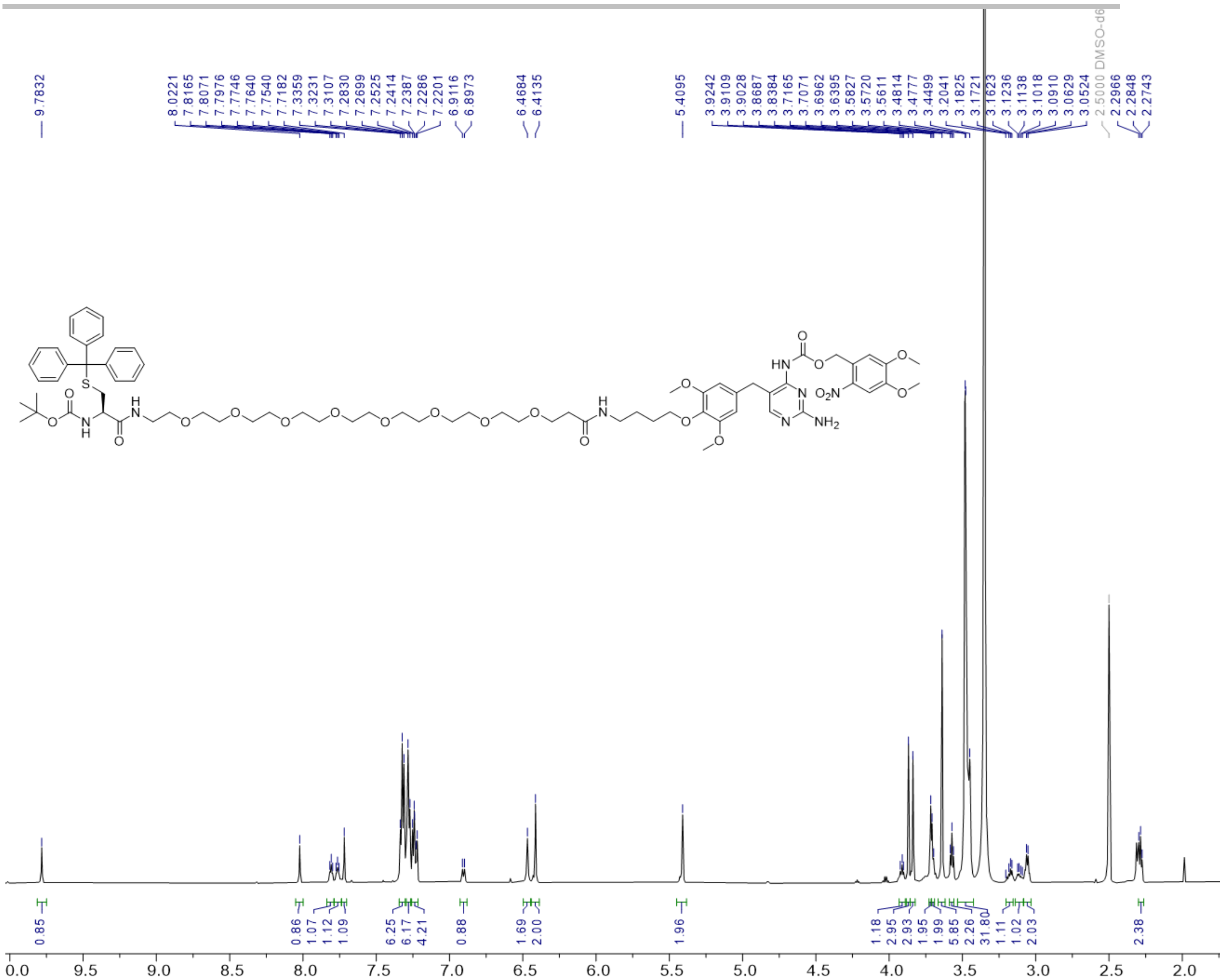

BocCys(Trt)-PEG₈-TMP(Novc): **^{13}C-NMR** (600MHz, DMSO-d^{6})

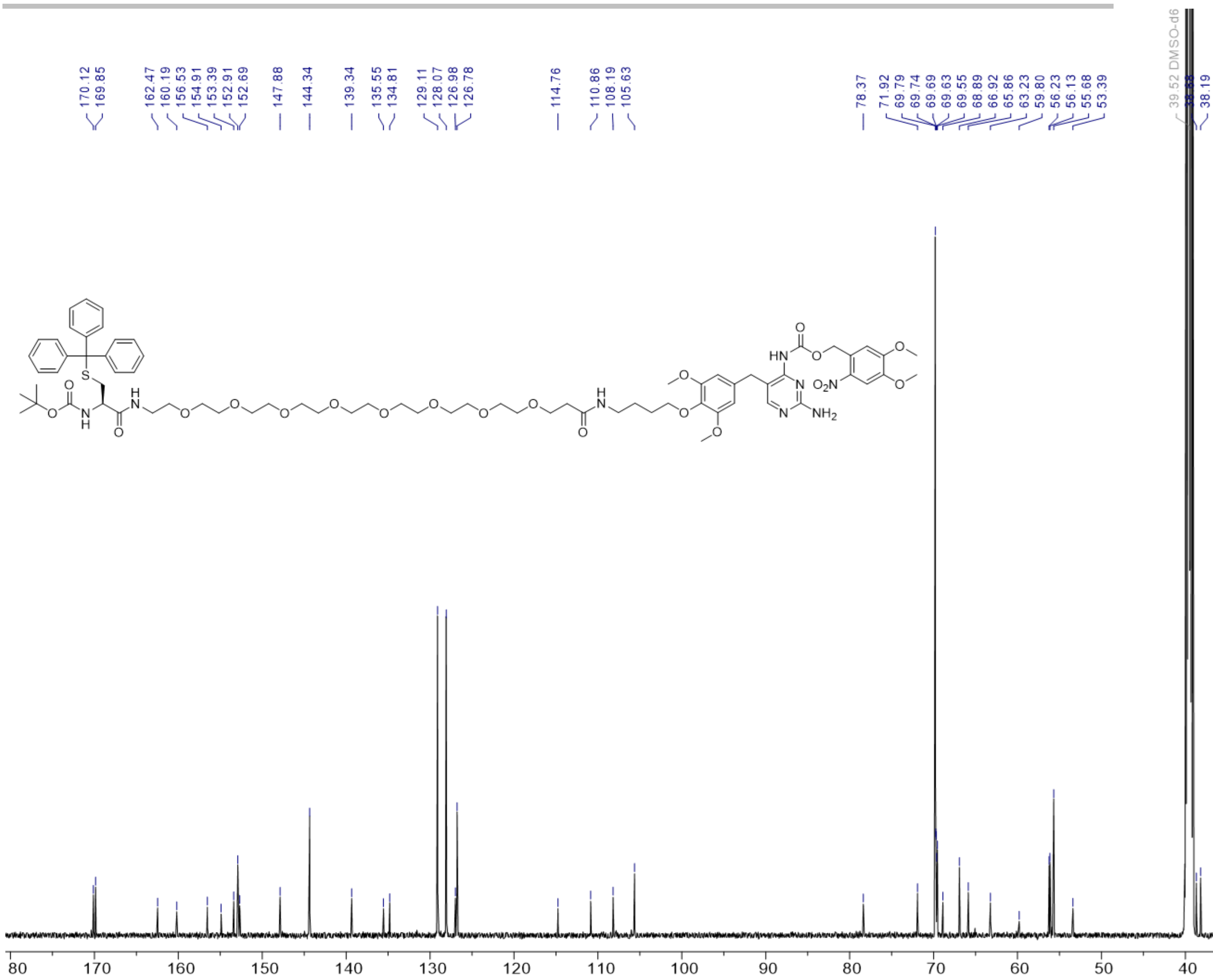

Cys-PEG$_8$-TMP(Novc): ^{1}**H-NMR** (600MHz, DMSO-d^6)

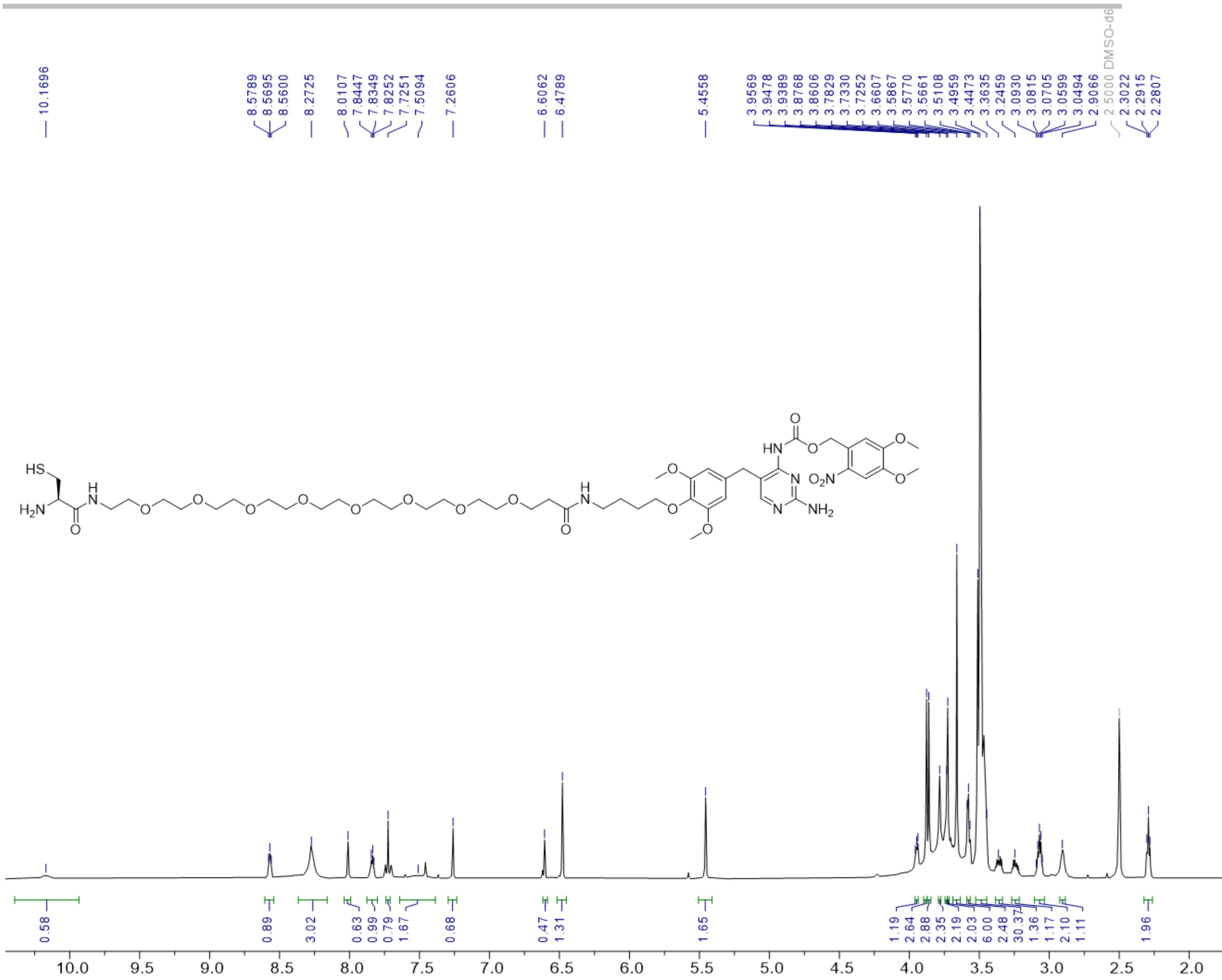

Cys-PEG$_8$-TMP(Novc): ^{13}**C-NMR** (600MHz, DMSO-d^6)

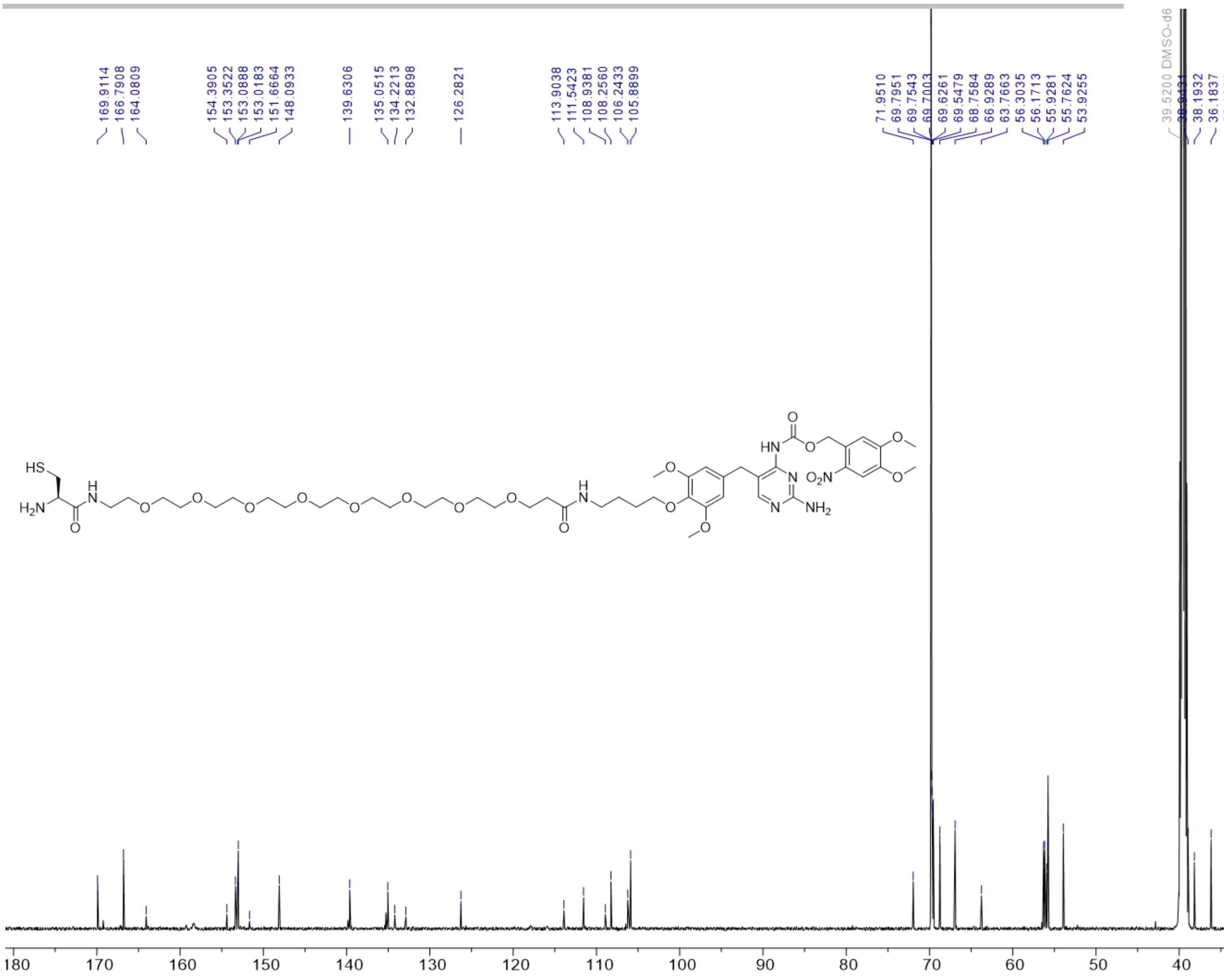

HR-MS Spectra

TMP-BuNHBoc

Single Mass Analysis
Tolerance = 3.0 PPM / DBE: min = -1.5, max = 50.0
Element prediction: Off
Number of isotope peaks used for i-FIT = 3

Monoisotopic Mass, Even Electron Ions
41 formula(e) evaluated with 1 results within limits (up to 50 closest results for each mass)
Elements Used:
C: 22-22 H: 10-35 N: 0-5 O: 0-7
8
0323-4-Z1 147 (0.828)

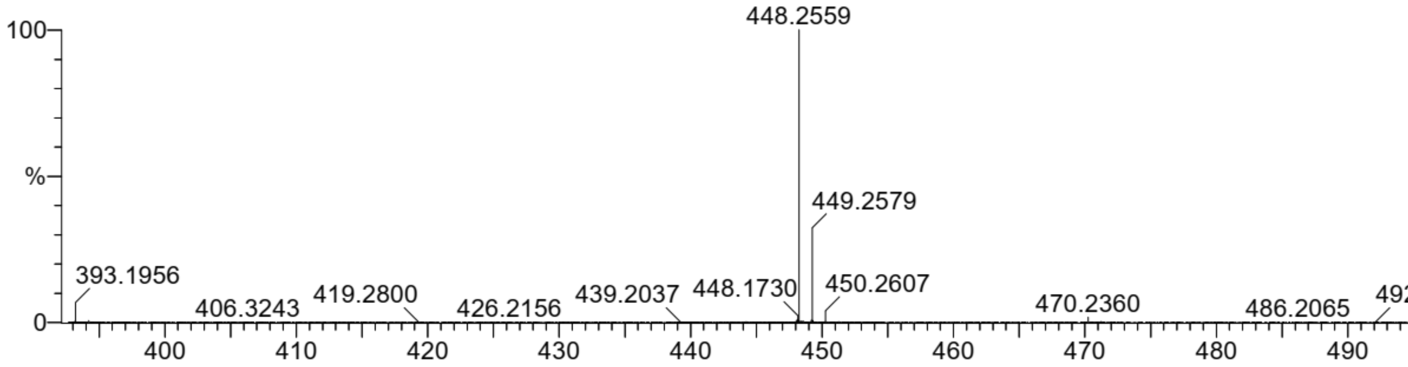

Minimum:				-1.5				
Maximum:		5.0	3.0	50.0				
Mass	Calc. Mass	mDa	PPM	DBE	i-FIT	Norm	Conf(%)	Formula
448.2559	448.2560	-0.1	-0.2	8.5	1195.4	n/a	n/a	C22 H34 N5 O5

N^4-NvocTMP-NHBoc

Single Mass Analysis
Tolerance = 3.0 PPM / DBE: min = -1.5, max = 50.0
Element prediction: Off
Number of isotope peaks used for i-FIT = 3

Monoisotopic Mass, Even Electron Ions
108 formula(e) evaluated with 1 results within limits (up to 50 closest results for each mass)
Elements Used:
C: 32-32 H: 10-50 N: 0-7 O: 0-15
8
0323-4-Z2 164 (0.925)

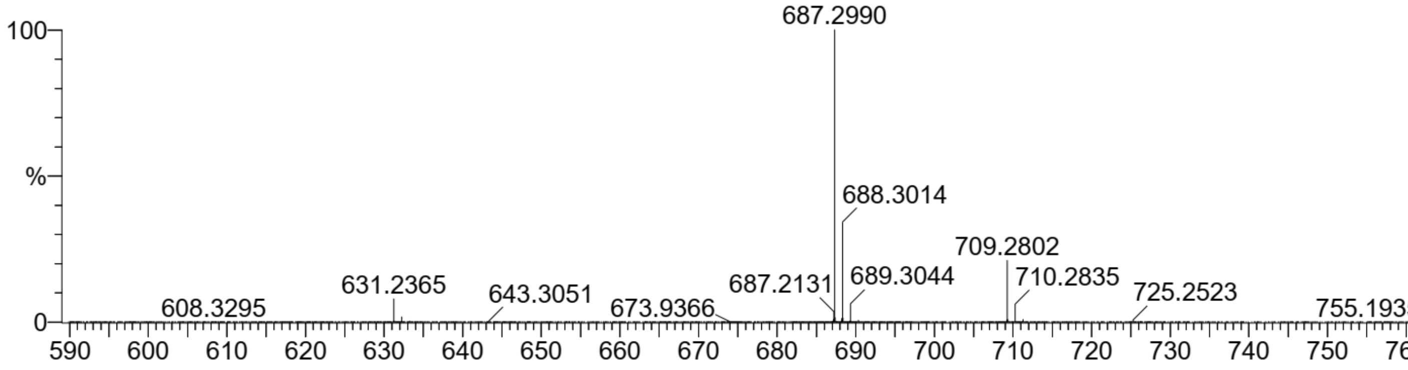

Minimum:				-1.5				
Maximum:		5.0	3.0	50.0				
Mass	Calc. Mass	mDa	PPM	DBE	i-FIT	Norm	Conf(%)	Formula
687.2990	687.2990	0.0	0.0	14.5	868.6	n/a	n/a	C32 H43 N6 O11

N^4-NvocTMP-PEG$_8$-NHBoc

Single Mass Analysis

Tolerance = 3.0 PPM / DBE: min = -1.5, max = 50.0
Element prediction: Off
Number of isotope peaks used for i-FIT = 3

Monoisotopic Mass, Even Electron Ions
165 formula(e) evaluated with 1 results within limits (up to 50 closest results for each mass)
Elements Used:
C: 51-51 H: 10-100 N: 0-7 O: 0-20
8
0323-4-Z4 166 (0.935)

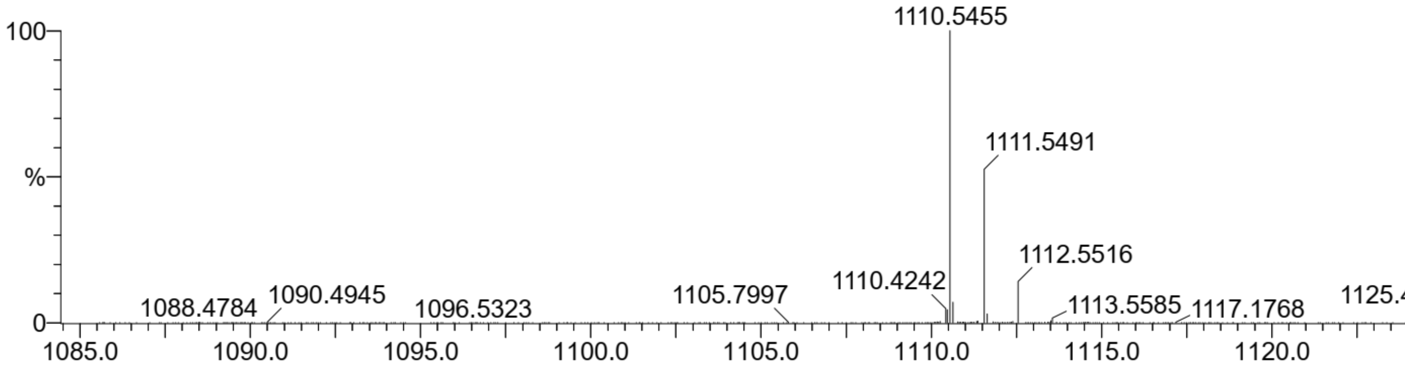

Minimum:				-1.5				
Maximum:		5.0	3.0	50.0				
Mass	Calc. Mass	mDa	PPM	DBE	i-FIT	Norm	Conf(%)	Formula
1110.5455	1110.5458	-0.3	-0.3	15.5	489.7	n/a	n/a	C51 H80 N7 O20

BocCys(Trt)-PEG8-TMP(Nvoc)

Single Mass Analysis

Tolerance = 3.0 PPM / DBE: min = -1.5, max = 50.0
Element prediction: Off
Number of isotope peaks used for i-FIT = 3

Monoisotopic Mass, Even Electron Ions
655 formula(e) evaluated with 1 results within limits (up to 50 closest results for each mass)
Elements Used:
C: 73-73 H: 10-100 N: 0-10 O: 0-21 S: 0-2
8
0323-4-Z7 135 (0.765)

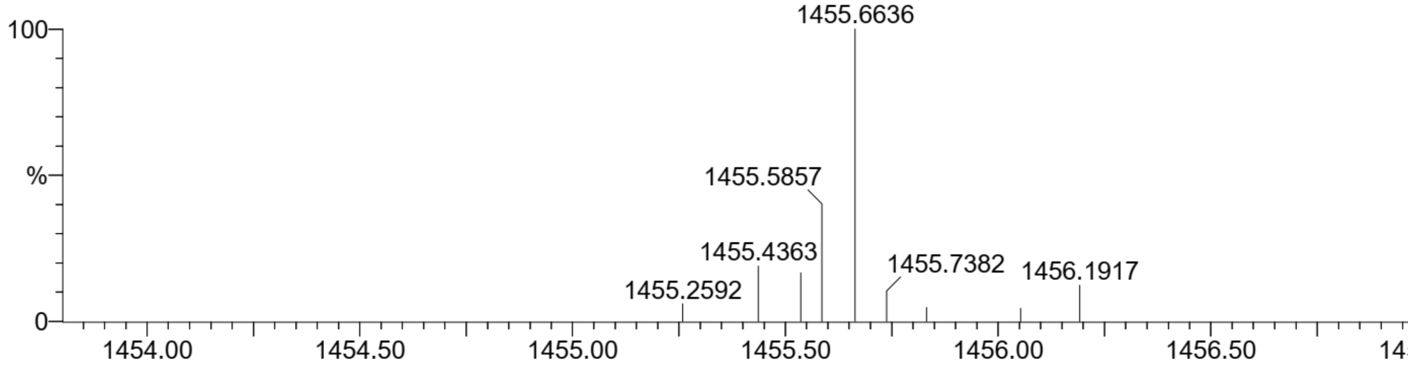

Minimum:				-1.5				
Maximum:		5.0	3.0	50.0				
Mass	Calc. Mass	mDa	PPM	DBE	i-FIT	Norm	Conf(%)	Formula
1455.6636	1455.6645	-0.9	-0.6	28.5	56.8	n/a	n/a	C73 H99 N8 O21 S

Cys-TMP(Nvoc)

Single Mass Analysis
Tolerance = 3.0 PPM / DBE: min = -1.5, max = 50.0
Element prediction: Off
Number of isotope peaks used for i-FIT = 3

Monoisotopic Mass, Even Electron Ions
701 formula(e) evaluated with 1 results within limits (up to 50 closest results for each mass)
Elements Used:
C: 49-49 H: 10-100 N: 0-10 O: 0-21 S: 0-2
8
0323-4-Z8 41 (0.248)

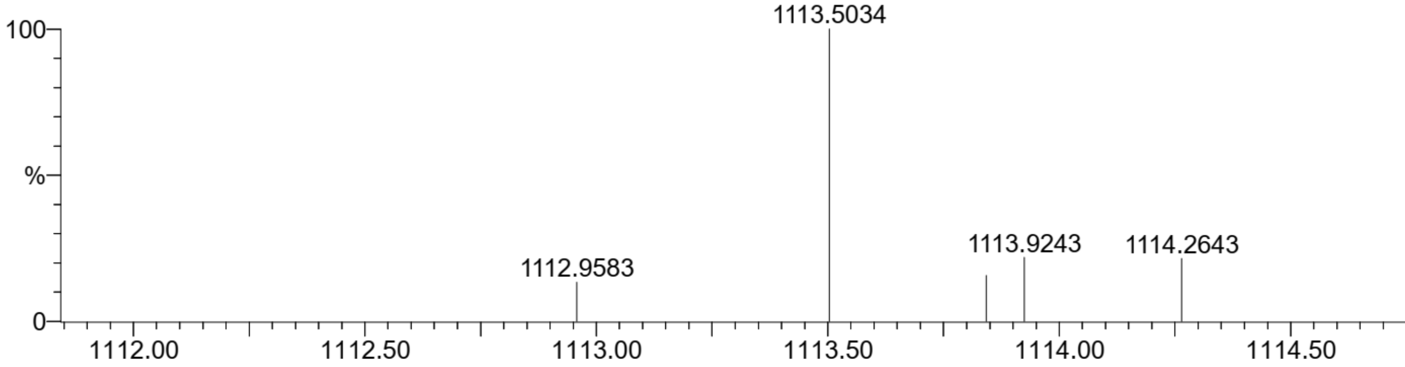

	Mass	Calc. Mass	mDa	PPM	DBE	i-FIT	Norm	Conf(%)	Formula
Minimum:					-1.5				
Maximum:			5.0	3.0	50.0				
	1113.5034	1113.5026	0.8	0.7	15.5	46.8	n/a	n/a	C49 H77 N8 O19 S

Author Contributions

Data curation: C. Zhou: lead contribution in data curation, validation and formal analysis; H. He: supporting contribution in data curation, validation and formal analysis; X. Chen: lead contribution in funding acquisition, investigation, projection administration and writing of original draft.
